# Development of Fluorogenic Probes to Detect Glycan-Degrading Enzymes in Bacteria

**DOI:** 10.1021/acsinfecdis.6c00347

**Published:** 2026-07-20

**Authors:** Esteban Tarazona Guzman, Ankita Paul, Karen D. Moulton, Daniel E. Calles-Garcia, Maren Cooper, Lucas J. DiCerbo, Soumyakanta Maji, Elizabeth A. Stemmler, Suvarn S. Kulkarni, Danielle H. Dube

**Affiliations:** † Department of Chemistry & Biochemistry, 2050 Bowdoin College, 6600 College Station, Brunswick, Maine 04011, United States; ‡ Department of Chemistry, 29491 Indian Institute of Technology Bombay, Powai, Mumbai 400-076, India

**Keywords:** glycosidase, fluorogenic glycoside, bacterial glycan, resorufin, activity-based protein profiling

## Abstract

Bacterial fitness and survival depend on the biosynthesis and maintenance of cell-envelope glycans. Bacterial glycans are composed of over 700 monosaccharides, and their construction, tailoring, recycling, and degradation require the choreographed action of myriad glycosyltransferases and glycosidases. Because of the structural diversity and complexity of bacterial glycans, coupled to limited methods to profile bacterial glycosidase activity, the function and existence of glycosidases that degrade most bacterial glycans are unknown. Taking a cue from the successful use of fluorogenic glycosides based on ubiquitous monosaccharides to detect glycosidase activity in cells and tissues, we sought to expand this approach to detect glycosidase activity in bacteria. Fluorogenic probes based exclusively on bacterial amino and deoxy sugars *N*-acetyl-d-fucosamine and 2,4-diacetamido-2,4,6-trideoxy-d-galactose were developed, and their activation was directly compared to a d-glucose-based fluorogenic probe in bacterial and mammalian cells. The species-selective utilization of these glycosidase probes, coupled to the difference in relative magnitude of glycosidase activity observed, provides important insights into these relatively unexplored glycan-processing enzymes. The use of these fluorogenic probes expands the toolkit for detecting glycan-degrading enzymes and offers an approach to enhancing a broader understanding of bacterial glycan metabolism and its diversity across different species.

## Graphical abstract

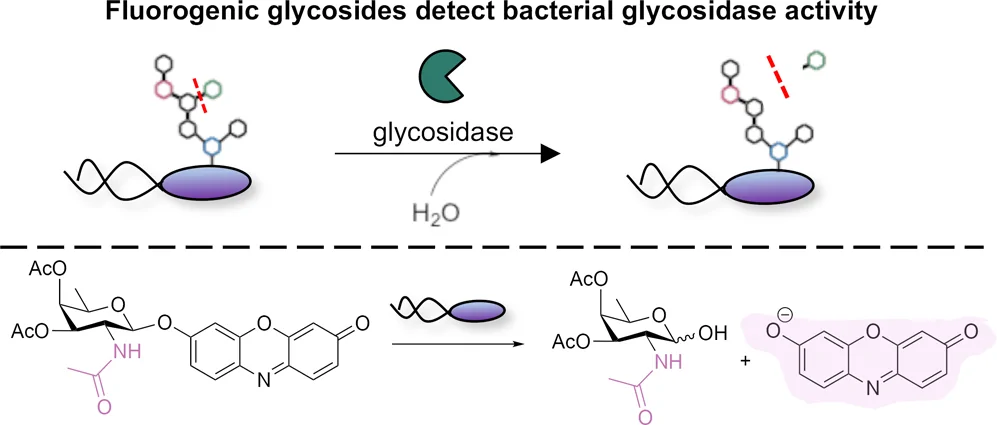

Unlike the biosynthesis of proteins and nucleic acids, which relies on a small number of highly conserved enzymes (e.g., ribosomes, polymerases) to construct molecules encoded by a genetic template, the biosynthesis of glycans relies on large families of glycan-processing enzymes acting in choreographed steps to build higher-order glycans that are not encoded by a template. Among these families of glycan-processing enzymes, glycoside hydrolases (GHs), also called glycosidases, catalyze the hydrolysis of glycosidic bonds (Figure 1A) to trim, tailor, recycle, and forage glycans.^1^ GH activities are essential for proper glycan biosynthesis and degradation, attributes that are critical for cell and organismal fitness. On the basis of bioinformatic studies using the carbohydrate active enzyme (CAZy) classification system, there are currently 189 GH families, with each family composed of hundreds of glycosidases.^2,3^ In all, nearly 1.8 million individual glycosidase genes have been identified to date.^2,3^ Given the actively expanding database and expansive classes of glycan-processing enzymes, assigning their biological activity is a considerable undertaking.^4^ Therefore, it is important to develop and expand the array of probes available to detect and characterize GH activity.

**Figure 1:**
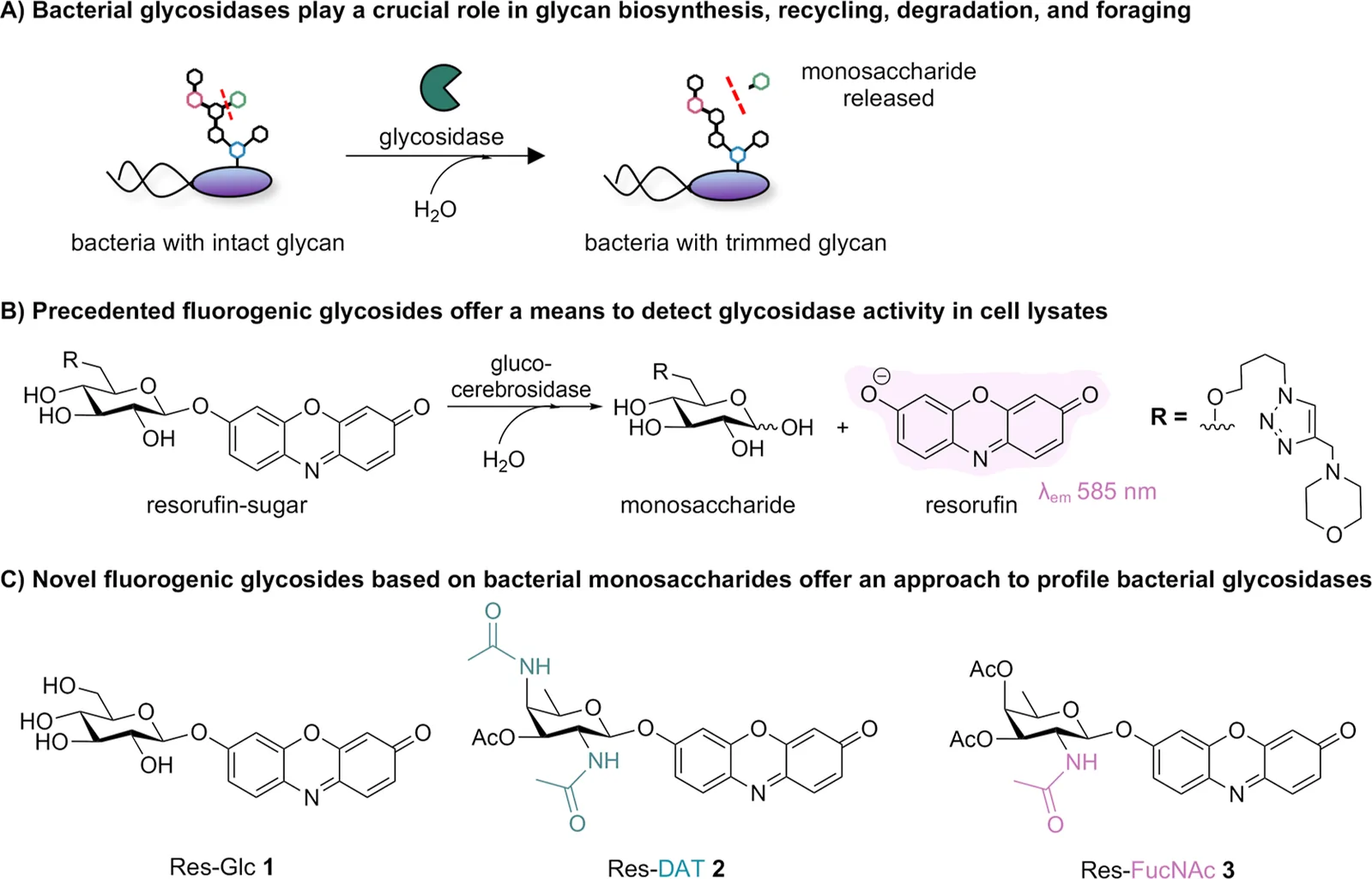
Bacterial glycosidases catalyze the hydrolysis of glycans to release monosaccharides and yield trimmed glycans, and fluorogenic glycosides serve as activity-based probes to facilitate glycosidase detection. (A) Bacterial glycosidases play a crucial role in glycan biosynthesis, recycling, degradation, and foraging. (B) Precedented fluorogenic glycosides offer a means to detect glycosidase activity in cell lysates. For example, resorufin-β-d-hexopyranoside analogs detect glucocerebrosidase activity in mammalian cell lysates and tissue homogenates by producing fluorescent resorufin following glycosidase-catalyzed hydrolysis of the glycosidic bond. (C) To expand upon previously reported fluorogenic glycosides based on ubiquitous monosaccharides such as resorufin-β-d-glucopyranoside (Res-Glc **1**), novel fluorogenic glycosides resorufin-β-d-DATDG (Res-DAT **2**) and resorufin-β-d-FucNAc (Res-FucNAc **3**) were developed based on the exclusively bacterial monosaccharides DATDG and FucNAc, respectively.

Fluorescence-based formats have opened the door to assigning GH activity for an assortment of substrates.^5–7^ For example, Vocadlo and co-workers developed fluorogenic glycoside hydrolase-activatable substrates that, upon recognition and cleavage by glucocerebrosidase, become fluorescent (Figure 1B).^8^ These glycosidase probes provide a means to screen glucocerebrosidase activity and its malfunction in Gaucher’s disease and Parkinson’s disease. More broadly, fluorogenic glycoside probes based on ubiquitous monosaccharides (e.g., glucose, fucose, GlcNAc), such as resorufin-β-d-glucopyranoside (Figure 1C, Res-Glc **1**), have facilitated screening of glycosidase activities present in systems ranging from human cells to bacteria to plants.^9–11^ Fluorogenic glycosidase probes have led to the identification of species-selective GH activities,^12^ information which in turn has set the stage for the development of GH inhibitors.^6,13^ Recent advances in the development of glycosidase probes have furthered our ability to detect glycosidases in real time and assign activity to the specific glycosidase that is responsible.^14^ Forster resonance energy transfer (FRET)-based probes have been deployed to visualize glycosidase activity in fixed tissue cross sections,^15^ and activity-based probes have been designed to covalently trap glycosidases for enrichment and identification.^11^

While glycosidase probes based on ubiquitous monosaccharides are poised to offer incisive insight into glycosidase structure and function, the knowledge learned from existing probes does not adequately capture the enormous amount of glycan structural diversity and utilization across the bacterial domain.^16^ In contrast to mammalian glycosidases, which have evolved relatively narrow substrate selectivity due to their specialized roles in glycan tailoring, the breadth of bacterial lifestyles and the range of niches that bacteria occupy (e.g., the gut microbiome, the soil) have led to the evolution of bacterial glycosidases that forage a diverse array of glycans with comparatively broad substrate specificity. Withers and co-workers recently utilized thioglycoside reporters to discover novel mechanisms used by gut bacteria glycosidases to degrade a broad array of carbohydrate substrates and thrive in this niche.^17^ In addition to the distinctive substrate profile and mechanisms utilized by bacterial glycosidases, the sheer complexity of bacterial glycans, which are collectively composed of more than 700 distinct monosaccharide building blocks,^18^ further complicates the study of glycans and their degradation in bacterial systems. The development of a small panel of activity-based probes based on bacterial glycans has provided robust and tractable bioassays to detect and assign the activity of select bacterial glycosidases. For example, custom-made probes based on *Pseudomonas aeruginosa* biofilm pentasaccharides^19^ and the biofilm polymer poly *N*-acetylglucosamine^20^ have enabled focused studies on biofilm modulation and inhibition in select pathogenic bacteria.^21^

Although bacteria utilize a plethora of distinctive, exclusively bacterial monosaccharides in glycan biosynthesis, to the best of our knowledge, none of these monosaccharides have been chemically modified into glycosidase probes. Herein, we report an expedient route to access resorufin-based analogs of the rare deoxy amino bacterial sugars d-2,4-diacetamido-2,4,6-trideoxy galactose (d-DATDG) and *N*-acetyl d-fucosamine (d-FucNAc) (Figure 1C, compounds **2** and **3**) and evaluate them as fluorogenic probes to detect glycosidase activity in a range of bacteria. As described below, the activation of fluorogenic probes based on these bacterial sugars was compared directly to the activation of resorufin-β-d-glucopyranoside (Res-Glc **1**; Figure 1C). Probe activation was explored in cells and lysates from human gastric adenocarcinoma cells that are presumed to exhibit minimal processing of bacterial glycans, commensal gut bacteria that are known to forage dietary monosaccharides, and stomach bacteria that exhibit more limited carbohydrate utilization. The species-selective utilization of these glycosidase probes, coupled to the difference in relative magnitude of glycosidase activity observed, provides important insight into these relatively unexplored glycan-processing enzymes. This work opens the door to identifying novel glycosidases in a range of bacteria, determining their substrate specificity and mechanisms of action, and screening for small molecule inhibitors that disrupt glycosidase function in a species-selective manner.

## Results and Discussion

### Design and Synthesis of Fluorogenic Resorufin Glycosides

Activity-based protein profiling has proved invaluable in a range of systems toward identifying enzymes responsible for biosynthetic transformations.^22^ Here, we sought to build upon activity-based approaches employed to detect and study glycoside hydrolases by developing probes based on bacterial monosaccharides rather than on ubiquitous monosaccharides. Inspired by fluorogenic glycoside hydrolase probes including commercially available resorufin β-d-glucopyranoside^23^ (Res-Glc **1**; Figure 1C), resorufin β-d-galactopyranoside,^24^ and derivatives (Vocadlo and coworkers),^8^ we designed resorufin d-glycosides based on the rare bacterial monosaccharide scaffolds DATDG and FucNAc (Figure 1C, compounds **2** and **3**). As a primary design element, these analogs contain fluorogenic resorufin, which becomes brightly fluorescent upon hydrolysis from the glycoside and has optical properties (λ_ex_ 571 nm, λ_em_ 585 nm) well suited to detection in cells. As a secondary design element, acetyl groups were installed to mask hydrophilic hydroxyl moieties and ensure efficient uptake by bacterial and mammalian cells to facilitate live cell studies.^25,26^ Following probe uptake into cells, acetyl protecting groups are hydrolyzed by cytosolic esterases to yield unmasked hydroxyl groups that can be recognized as glycosidase substrates.

To access novel fluorogenic analogs resorufin-d-DATDG (Res-DAT **2**) and resorufin-d-FucNAc (Res-FucNAc **3**), we adapted and built on our previous strategies. We relied on the protocol reported by Kulkarni and co-workers to access functionalized thioglycoside analogs of DATDG and fucosamine. We synthesized analogs **2** and **3** by adapting our previous strategy, using a regioselective displacement of pyranosidic C-2/C-4 bistriflates with azide or nitrite nucleophiles to access functionalized rare sugar thioglycosides (bacillosamine, DAT, and fucosamine).^27,28^ Initially, the thioglycoside **4** was activated using NIS and subjected to coupling with commercially available resorufin-Na salt using TMSOTf or TfOH, but it failed to give the coupled product (Scheme 1, entry 1, Table 1). In the next attempt, the thioglycoside **4** was converted to its corresponding hemiacetal using NBS and further treated with CCl_3_CN and K_2_CO_3_ to give a more reactive DAT trichloroacetimidate **5** ^29^ (Scheme 1, entry 2, Table 1). To our dismay, no coupled product was observed even when glycosyl imidate **5** was coupled with resorufin sodium salt in the presence of TMSOTf/TfOH at varied temperatures. When thioglycoside **4** was converted to its glycosyl bromide using Br_2_ and CH_2_Cl_2_ (Scheme 1, entry 3, Table 1) and coupled with resorufin sodium salt in the presence of Lewis acid AgOTf, a faint fluorescent spot was observed on TLC, which corresponds to 10% of the desired fluorescent sugar **7** along with the hemiacetal of the corresponding donor. However, the yield of the glycosylation was improved to 52% with the use of Ag_2_CO_3_ as a promoter. The β-stereochemistry of the formed glycosidic linkage in **7** was confirmed from the appearance of the chemical shift of the anomeric proton at δ 4.98 Hz and the ^3^ *J*_(H1, H2)_ coupling constant of 8.1 Hz in the ^1^H NMR spectrum and δ 99.9 in the ^13^C NMR spectrum.

**Scheme 1:**
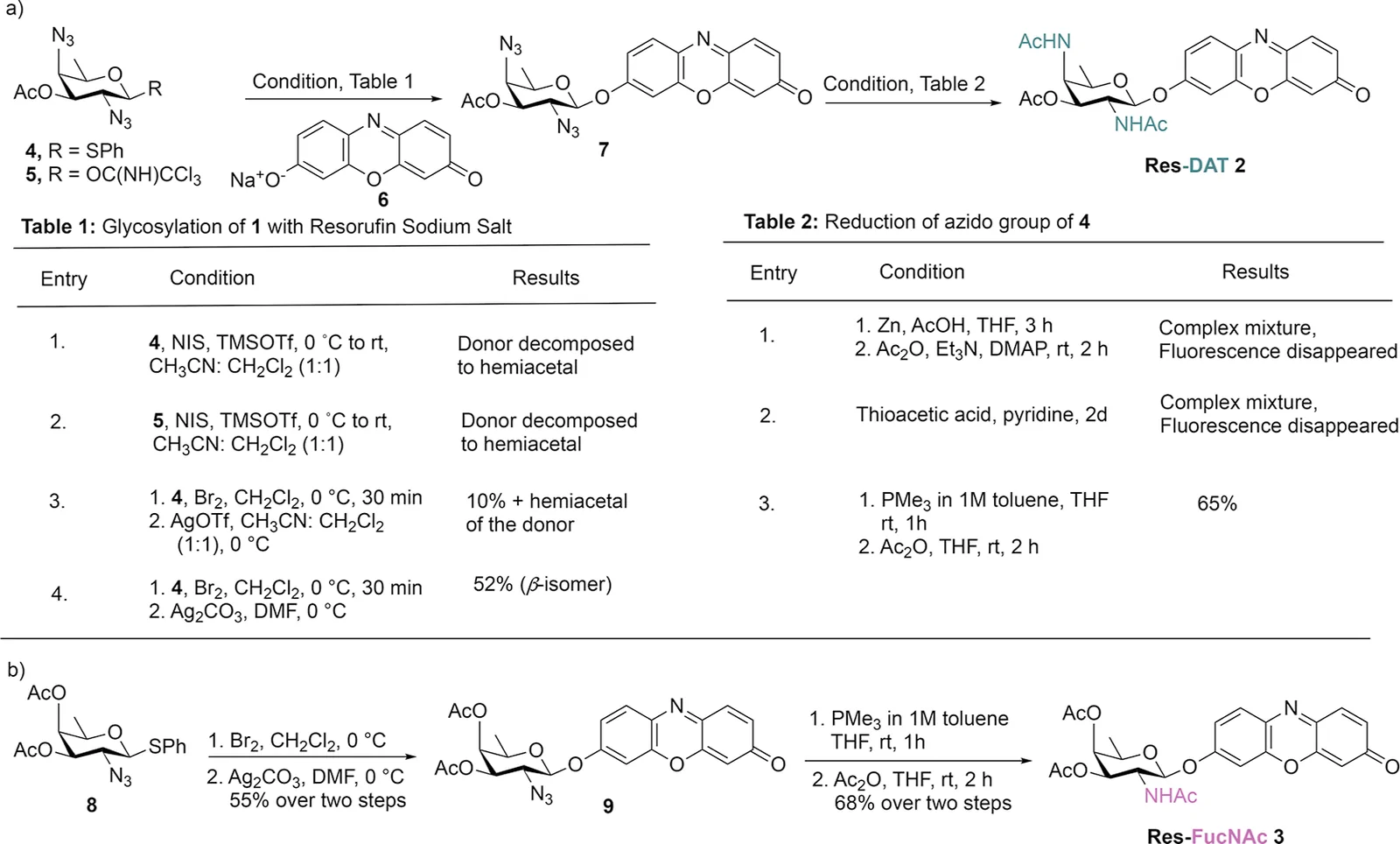
Synthesis of Rare Sugar Fluorogenic Analogs Res-DAT **2** and Res-FucNAc **3**

Post glycosylation, we turned our attention to the final conversion of the azide to NHAc. For this, we first tried metal-mediated conditions, i.e., Zn and AcOH for reduction of azide to amine, but at this stage, no polar fluorescent spot indicative of amine was observed on the TLC (Scheme 1, entry 2, Table 2). Also, the ^1^H NMR was complex and did not indicate the desired product formation. Milder conditions such as thioacetic acid and pyridine also failed to fetch the desired compound (Scheme 1, entry 3, Table 2). Finally, PMe_3_ in 1 M toluene could successfully reduce both C2 and C4 azides to their corresponding amine, which was then protected with acetate using Ac_2_O to give fluorescent DAT-resorufin derivative **2** (Scheme 1, entry 3, Table 1). Later, we followed a similar optimized condition to obtain FucNAc-resorufin derivative **3** from Fuc-thioglycoside **8**.

### Fluorogenic Resorufin Glycosides Are Activated by Glycosidases in Lysates and Cells

We first sought to assess fluorogenic glycoside activation in vitro using a commercially available glycosidase. We turned to the well-characterized enzyme β-glucosidase, which catalyzes the hydrolysis of terminal β-glucose from glycoconjugates. Briefly, we expected treatment of Res-Glc **1** with β-glucosidase to hydrolyze glucose from the probe and yield the fluorescent aglycone resorufin. By contrast, we did not expect β-glucosidase to recognize Res-DAT **2** or Res-FucNAc **3** as substrates due to the identity of the sugars in these probes and their acetylated state, both of which should preclude enzyme–substrate binding. To test this hypothesis, 200 μM resorufin glycosides **1**–**3** were incubated in McIlvaine buffer (pH 7) with or without 2 μM β-glucosidase for 30 min. In accord with expectation, incubation of Res-Glc **1** with β-glucosidase led to a dramatic increase in fluorescence intensity at 585 nm relative to incubation in buffer alone (Figure 2A). Conversely, minimal fluorescence was observed for Res-DAT **2** and Res-FucNAc **3** samples incubated with β-glucosidase, and the signal observed in these samples was on par with background fluorescence observed with the probes incubated in buffer alone (Figure 2A). High-performance liquid chromatography analysis confirmed that incubation of Res-Glc **1** with β-glucosidase led to the release of resorufin (Figure S1). These data confirm that fluorescence activation is due to specific glycosidase activity rather than nonspecific hydrolysis of the glycosidic bond. Moreover, these data indicate the relative stability of resorufin glycosides **1**–**3** in aqueous buffer in the absence of a matched glycosidase.

**Figure 2:**
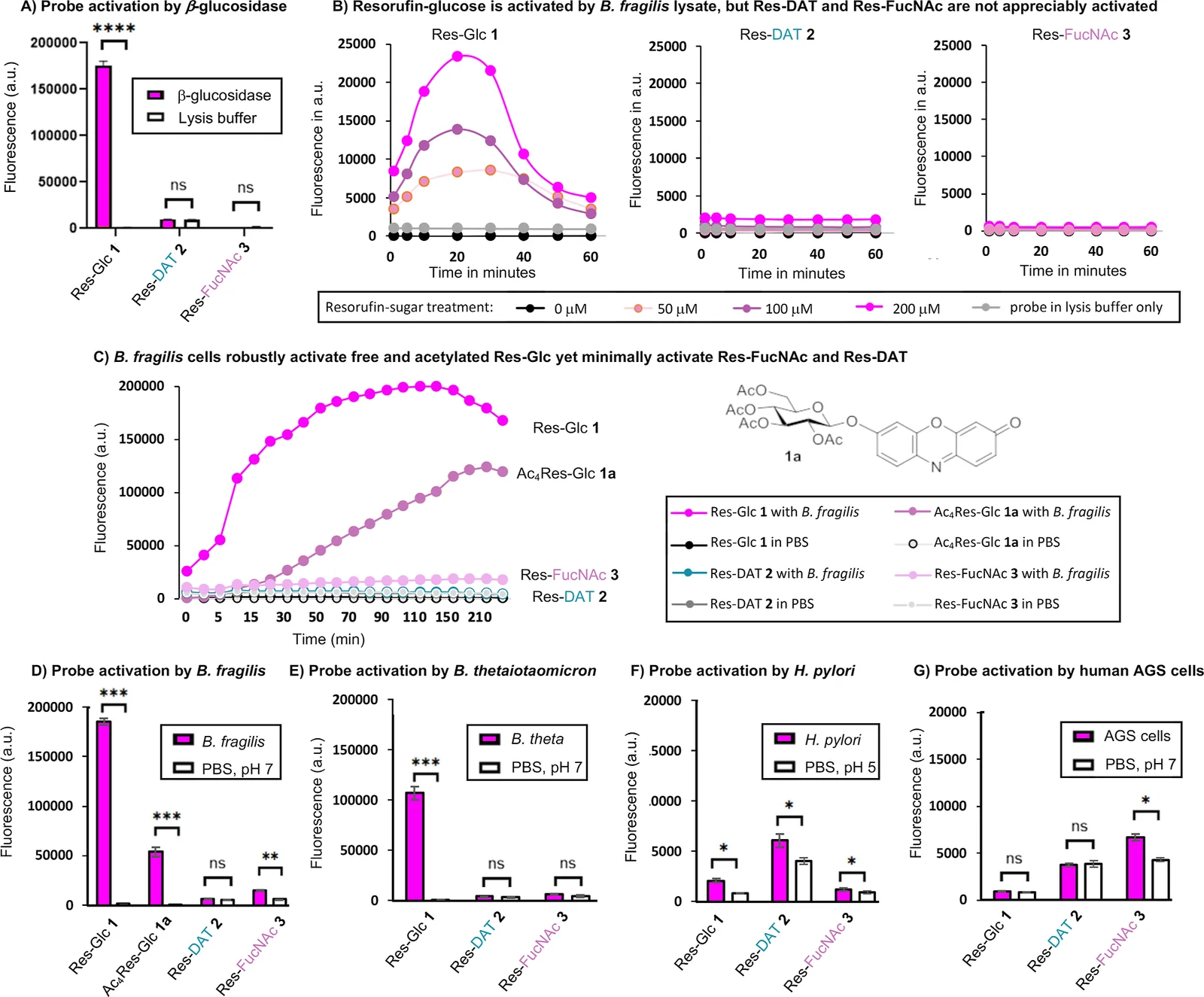
Fluorogenic resorufin glycosides are activated to varying extents by commercially available β-glucosidase, a range of bacterial cells, and mammalian cells. (A) The indicated resorufin glycosides (200 μM) were incubated in lysis buffer with or without β-glucosidase, and fluorescence was monitored at 585 nm after 30 min to detect glycosidase-catalyzed resorufin release. (B) *Bacteroides fragilis* lysate was incubated with resorufin glycosides at a range of concentrations (0–200 μM), and fluorescence was monitored at 585 nm over 60 min to detect glycosidase-catalyzed resorufin release. (C) Resorufin glycosides (200 μM) were incubated with live *B. fragilis* in PBS, and fluorescence at 585 nm was measured and compared to probes incubated in PBS alone over 4 h. (D–G) Resorufin glycosides (200 μM) were incubated in PBS containing (D) *B. fragilis*, (E) *Bacteroides thetaiotaomicron* (*B. theta*), (F) *Helicobacter pylori*, or (G) AGS cells for 60 min, and fluorescence at 585 nm was measured and compared to probes incubated in PBS alone during the same interval. Error bars represent technical replicates from triplicate samples. Data are representative of independent experiments (*n* = 3) that exhibited the same findings. Welch’s *t*-test was used to determine statistical significance between paired control and experimental samples (*P < 0.01; **P < 0.001; ***P < 0.0001; ****P < 0.00001; ns = nonsignificant).

We next sought to determine the optimal probe concentration and time for monitoring glycosidase activity with fluorogenic resorufin glycosides. For these experiments, we began with the commensal intestinal bacteria *Bacteroides fragilis* due to its role in degrading dietary carbohydrates with an array of glycosidases.^30^ *B. fragilis* lysates were incubated with 0–200 μM resorufin glycosides **1**–**3**, and fluorescence at 585 nm was monitored over 1 h and compared to the background fluorescence of probes in lysis buffer only. Res-Glc **1** samples exhibited significant fluorescence following incubation with *B. fragilis* lysate (Figure 2B). Fluorescence peaked following 20 min of incubation, was dependent on probe concentration, and was highest with 200 μM Res-Glc **1**. By contrast, there was relatively little background fluorescence observed in samples containing Res-Glc **1** in lysis buffer, and the low levels of background fluorescence did not change over time. These data align with reported β-glucosidase activity in *B. fragilis* and underscore probe stability in the absence of glycoside hydrolases. *B. fragilis* lysate led to significant activation of Res-Glc **1** yet exhibited minimal activation of Res-DAT **2** or Res-FucNAc **3** relative to buffer-only controls (Figure 2B). These data establish parameters for monitoring glycosidase activity and reveal clear substrate specificity for glycosidases in *B. fragilis* lysates.

We next assessed the extent to which fluorogenic resorufin glycosides were activated by glycosidases produced by live *B. fragilis*. For these experiments, we probed glycosidase activity in *B. fragilis* cells treated with 200 μM fluorogenic glycosides **1**–**3** and monitored fluorescence at 585 nm over 4 h relative to background fluorescence of the probe in phosphate-buffered saline (PBS) only. Curious about the potential impact of probe acetylation on the activation of fluorogenic glycosides, we prepared acetylated Res-Glc **1a** to directly compare its activation relative to the other probes (Figure 2C). Robust activation of both acetylated Res-Glc **1a** and hydroxylated Res-Glc **1** was observed in *B. fragilis* cells, with considerably more activation of these probes relative to Res-DAT **2** and Res-FucNAc **3** (Figure 2C). There was a lag in activation of acetylated Res-Glc **1a** and a lower maximum level of fluorescence activation relative to Res-Glc **1**, consistent with the dynamics of probe deacetylation prior to glycosidase-mediated hydrolysis. This time course experiment revealed that 60 min offers sufficient time to detect fluorogenic glycoside activation while accounting for probe deacetylation in live *B. fragilis*. Since both **1** and **1a** were able to report on glycosidase activity in *B. fragilis* cells, we opted to use Res-Glc **1** as a point of comparison for subsequent experiments.

Next, we sought to probe the extent of fluorogenic glycoside activation by glycosidases produced in a range of cell types. For these experiments, we probed glycosidase activity in cells treated with 200 μM fluorogenic glycosides **1**–**3** and monitored fluorescence after 60 min of incubation, with the rationale that cell-based experiments require slightly more time for probe activation than in lysates, where glycosidases are immediately able to access substrate. We probed glycosidase activity in two ubiquitous intestinal bacteria—*B. fragilis* and *Bacteroides thetaiotaomicron*—that are known to forage dietary glycans as part of their lifestyle strategy,^31^ the pathogenic bacterium *Helicobacter pylori* that colonizes the human stomach,^32^ and human gastric adenocarcinoma (AGS) cells. Consistent with probe activation following incubation with *B. fragilis* lysate activation, Res-Glc **1** and acetylated Res-Glc **1a** exhibited robust fluorescence following incubation with *B. fragilis* cells in PBS for 60 min (Figure 2D). *B. fragilis* cells led to low yet significant levels of Res-FucNAc **3** activation (Figure 2D; Figure S2A). Since *B. thetaiotaomicron* shares a similar lifestyle strategy to *B. fragilis*, we wondered if this bacterium’s glycosidase activity profile would mirror *B. fragilis*’s profile. Incubation of *B. thetaiotaomicron* with Res-Glc **1** led to a dramatic increase in fluorescence relative to cell-free controls (Figure 2E), suggesting this bacterium expresses glycosidases that recognize glucose-bearing glycosides as substrates. No appreciable activation of Res-DAT **2** or Res-FucNAc **3** was observed in the presence of *B. thetaiotaomicron* cells (Figure 2E; Figure S2B) or lysates (Figure S2F). *H. pylori* cells led to low-to-modest fluorescence activation of Res-Glc **1**, Res-DAT **2**, and Res-FucNAc **3** (Figures 2F and S2C). Lastly, AGS cells did not appreciably activate Res-Glc **1** or Res-DAT **2** but led to some fluorescence activation of Res-FucNAc **3** (Figures 2G and S2D) despite d-FucNAc’s absence from mammalian glycans. Similar trends in glycosidase activity and substrate selectivity were observed when resorufin glycosides **1**–**3** were incubated with lysates from these cells (Figure S2E–H). Taken together, these data align with reported β-glucosidase activity and offer insight into more specialized glycoside hydrolase recognition of glycans bearing exclusively bacterial sugars. Moreover, activation of novel fluorogenic glycoside probes by live cells and cell lysates validates their utility in probing glycosides based on bacterial glycans.

### Fluorogenic Resorufin Glycosides Facilitate Detection of Glycosidase Activity via In-Gel Fluorescence

Once glycoside hydrolase activity corresponding to fluorogenic activation was observed with substrates in a range of cells, we sought to pinpoint enzymes responsible for substrate activation by in-gel staining with these probes. Briefly, lysates from *B. fragilis* were resolved via native polyacrylamide gel electrophoresis, and in-gel staining of electrophoresed samples was performed with 2 μM Res-Glc **1** to detect proteins corresponding to fluorogenic activation.^7^ We electrophoresed commercially available β-glucosidase and bovine serum albumin (BSA) as positive and negative controls, respectively, to analyze alongside *B. fragilis* lysates. In-gel fluorescence detection at 568 nm revealed an intense signal corresponding to a single low molecular weight protein in the negative control, which should lack glucosidase activity; this low molecular weight species was therefore interpreted as a nonspecific signal (Figure 3A). Several highly fluorescent high molecular weight species were present in the β-glucosidase positive control yet absent from the negative control (Figure 3A), consistent with the expected β-glucosidase activity in this sample. Despite the large number of proteins observed in *B. fragilis* lysate by Coomassie blue staining of electrophoresed proteins (Figure 3B), a single punctate fluorescent band was observed by in-gel fluorescence analysis of this sample (Figure 3A, asterisk), suggesting that resorufin-glucose activation can be pinpointed to a single high-molecular-weight protein in *B. fragilis*. In-gel fluorescence of *B. fragilis* samples with Res-DAT **2** and Res-FucNAc **3** led to no appreciable fluorescence activation (Figure S3), lending further confidence that *B. fragilis* glucosidases discriminate among substrates. In-gel fluorescence of *H. pylori* lysates incubated with resorufin glycosides **1**–**3** led to negligible fluorescence activation (Figure S3), likely due to the lower relative magnitude of glycosidase activity observed in this bacterium (Figures 2E and S2). The *B. fragilis* protein band that fluoresces in the Res-Glc **1** treated gel was excised to identify a likely glycosidase corresponding to this activation event. Briefly, in-gel digestion with trypsin followed by LC–MS/MS analysis of peptides derived from intact proteins led to the identification of the CAZy-annotated^33^ *B. fragilis* β-glucosidase BglX alongside a variety of housekeeping and contaminant proteins that have no proposed glycosidase activity. These findings suggest that BglX is likely the glycosidase responsible for Res-Glc **1** activation, consistent with reported activity and substrate specificity of this enzyme.^30^ This proof-of-principle experiment suggests that in-gel fluorescence with fluorogenic resorufin glycosides offers inroads to identify novel glycosidase activities and assign these enzymes to their substrates.

**Figure 3:**
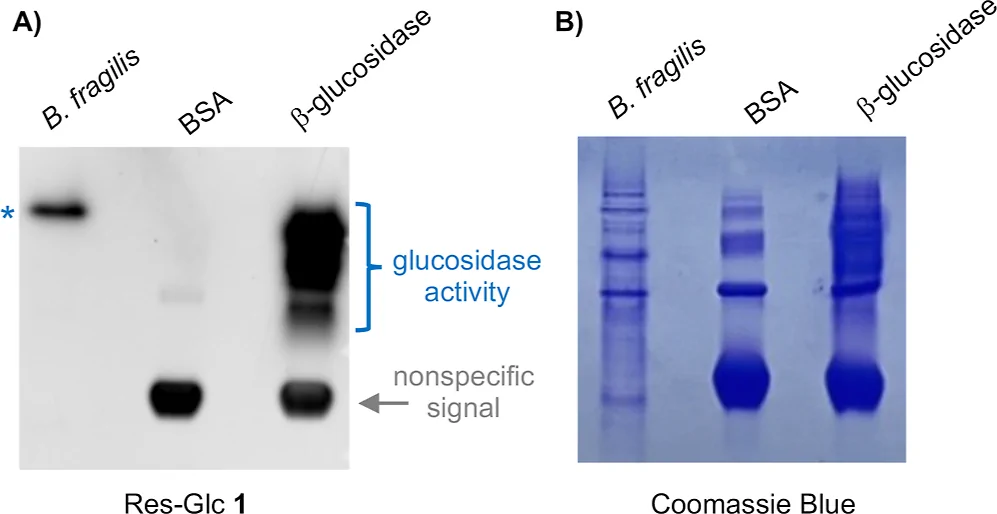
Resorufin-glucose activation by *B. fragilis* lysate can be pinpointed to one protein. (A,B) *B. fragilis* lysate, the negative control bovine serum albumin (BSA), and the positive control β-glucosidase were separated using native PAGE and (A) incubated with Res-Glc **1** (2 μM) for 15 min prior to measuring in-gel fluorescence at 568 nm to visualize glycosidase activity or (B) stained using Coomassie blue to visualize protein loading. An asterisk marks the band that was excised for trypsinization and mass spectrometry analysis. Data are representative of replicate experiments (*n* = 2).

## Discussion

Activity-based probes offer a powerful approach for detecting specific types of enzymatic activity in cellular samples and identifying enzymes that act on a particular substrate. Indeed, since the pioneering development of activity-based probes by Cravatt,^34^ Bogyo,^35^ and others, a plethora of probes have been developed to report on different classes of enzyme activity,^22,36,37^ including glycoside hydrolases.^13^ Fluorogenic, ratiometric, and activity-based probes have provided high-throughput assays to detect and characterize select classes of glycosidase activity^14^ and begin the daunting task of assigning biological activity to the vast number of glycosidases that are predicted on the basis of bioinformatic evidence. Continued expansion of the suite of glycosidase probes available and application of glycosidase probes to a broader range of cell types offer paths to study glycosidase families whose existence is postulated from sequence analysis alone. Here, we developed fluorogenic resorufin glycosides based on exclusively bacterial amino deoxy sugars and used them in gastrointestinal bacteria involved in human health and disease to pan for novel glycosidase activities. These compounds expand the chemical glycobiology toolkit, provide first experimental evidence of previously unreported glycosidase activities in bacteria, and offer information about patterns of glycosidase activity across a range of cell types.

Commercially available Res-Glc **1** served as a valuable point of comparison for these studies, as this fluorogenic glycoside is based on the ubiquitous monosaccharide glucose. While Res-Glc **1** was robustly activated by glycosidases in the commensal gut bacteria *B. fragilis* and *B. thetaiotaomicron*, this probe was activated at low levels by the stomach pathogen *H. pylori* and minimally by AGS cells (Figures 2D–G and S2). By contrast, *H. pylori* incubated with Res-Glc **1**, Res-DAT **2**, and Res-FucNAc **3** led to modest probe activation, indicating the presence of relatively inefficient or nonspecialized glycosidases that recognize these substrates. Overall, the extent of fluorogenic glycoside activation in our study varied across cells and in a substrate-dependent manner, with different activation patterns observed between cell types. Patterns of probe activation may reveal which GH activities are required for cell fitness for divergent biological niches and lifestyles. For example, since *H. pylori* effectively incorporates DAT and FucNAc analogs in metabolic labeling experiments,^38^ even modest levels of glycoside hydrolase activity recognizing these sugars may aid this bacterium in glycan tailoring and recycling. By contrast, since DAT or FucNAc are not common components of dietary glycans nor components of *Bacteroides sp*. or AGS glycans, *B. fragilis*, *B. thetaiotaomicron*, and AGS cells may not have evolved glycosidases that efficiently recognize glycosides bearing these distinctive bacterial monosaccharides as substrates. This study provides evidence for glycosidases that recognize unusual bacterial monosaccharides in select bacteria and sets the stage for further panning and characterization of the responsible enzymes.

In a proof of principle experiment, we demonstrated that an in-gel fluorescence assay with fluorogenic glycoside Res-Glc **1** allowed the visualization and identification of β-glucosidase activity in *B. fragilis* lysate (Figure 3A). This finding aligns with previous reports in which activity-based probes were used to identify enzymes, including glycosidases, responsible for probe activation.^39^ The CAZy database^33^ offered a valuable resource for cross-checking the array of proteins identified by mass spectrometry and helping to pinpoint the putative *B. fragilis* β-glucosidase. This approach could be extended to identify glycoside hydrolases corresponding to novel glycosidases that hydrolyze Res-Glc **1**, Res-DAT **2**, and Res-FucNAc **3**. Once candidate glycoside hydrolases are identified, they can be biochemically purified and validated using in vitro enzyme kinetics experiments with fluorogenic probes (e.g., Vocadlo and co-workers).^8^ Thus, coupling these fluorogenic glycosides to in-gel activation assays offers a path to yield first insights into novel glycoside hydrolases in bacterial systems and to assign biochemical functions to putative glycosidase genes.

Probe acetylation was used as a design parameter to increase the likelihood of fluorogenic glycosides Res-DAT **2** and Res-FucNAc **3** crossing the lipophilic membrane and having utility in live-cell experiments. Previous studies suggest that there are likely sufficient levels of carbohydrate esterase activity in *B. fragilis*, *H. pylori*, and AGS cells to hydrolyze acetyl-protecting groups and transform acetylated probes into free sugars.^26^ The observed fluorescent activation of acetylated fluorogenic probes **2** and **3** in selected cells and lysates indicates that these probes were utilized as substrates in these cells. In contrast to our expectation, free Res-Glc **1** exhibited superior cellular activation in *B. fragilis* than acetylated Res-Glc **1a** (Figure 2C). This observation suggests that probe acetylation is not an optimal design parameter in *B. fragilis* for glycosidase probes. Moreover, this observation indicates that free Res-Glc **1** is transported across the cell envelope. Nonetheless, since both free and acetylated probes **1** and **1a** were able to report on glycosidase activity in *B. fragilis* cells, probe acetylation does not pose an impediment for the detection of glycosidase activity in this bacterium. Probing the extent to which acetylation impacts probe uptake, use, and activation in a range of cell types is a worthwhile future direction.

While acetylation as a design parameter may be a strength in some contexts, it poses a limitation under certain circumstances. For example, cells that lack sufficient esterase activity would be unable to unmask the underlying hydroxyl groups of acetylated probes **2** and **3**; probes that remain acetylated are unlikely to be recognized as substrates by glycosidases, rendering these probes unusable in cells with low esterase activity. Moreover, acetylated probes **2** and **3** have limited utility in native PAGE assays since esterase activity and glycosidase activity would need to comigrate in the gel for probe deacetylation to yield a glycosidase substrate at the precise location where a glycosidase is in a gel. Indeed, Res-DAT **2** and Res-FucNAc **3** did not yield any detectable fluorescence activation via in-gel fluorescence assays with *B. fragilis* or *H. pylori* lysates (Figure S3), which could be due in part to probe acetylation posing a liability for gel-based activation studies. Access to both hydroxylated and acetylated versions of fluorogenic glycoside probes would overcome the limitations posed by acetylation. In addition to probe acetylation potentially serving as a barrier for in-gel fluorescence detection, low signal-to-noise ratios for select probe-bacteria pairs (e.g., Res-FucNAc **3** in *H. pylori*) diminish the likelihood of pinpointing enzymatic activity.

These studies were focused on accessing glycosidase probes based on bacterial glycan epitopes and validating fluorogenic glycosides in a small number of bacterial and one human cell line. Novel probes **2** and **3** will open the door to studying glycan processing and degradation in a broad range of species, probing glycan tailoring in complex settings including mixed microbial populations, identifying cell-selective activities that could form the basis of diagnostics, assigning biological activities to the responsible glycosidase, and screening for small-molecule inhibitors that disrupt glycosidase function in a species-selective manner. Access to these probes will set the stage for determining the importance of specific glycosidase activities in a variety of bacterial lifestyles, serve as a template for new probe designs to further broaden the toolkit, and provide leads to identify new mechanisms for foraging, degrading, and recycling glycans.

## Conclusion

This study led to the design and synthesis of novel fluorogenic glycosides based on exclusively bacterial sugars, validation of these probes in a small number of bacterial and human cells, and the use of these probes to detect and pinpoint glycosidase activity via in-gel probe activation. Expansion of the suite of tools suited to detect and study glycosidases in bacteria will open the door to tracking glycan tailoring in real time and in complex settings, identifying cell-selective activities that could form the basis of diagnostics, assigning biological activities to the responsible glycosidases, and screening for small-molecule inhibitors that disrupt glycosidase function in a species-selective manner. Ultimately, access to these probes will set the stage for determining the importance of specific glycosidase activities in a variety of bacterial lifestyles, serve as a template for new probe designs to further broaden the glycochemistry toolkit, and provide leads to identify new mechanisms for foraging, degrading, and recycling glycans.

## Methods

### Materials

All organic reagents were obtained from MilliporeSigma. *H. pylori* strain G27 was obtained from Dr. Manuel Amieva (Stanford University). *B. fragilis* (ATCC 23745), *B. thetaiotaomicron* (ATCC 700349), and AGS cells (ATCC CRL-1739) were purchased from ATCC and cultured according to the manufacturer’s instructions. Resorufin-β-d-glucopyranoside **1** and β-glucosidase from almonds were purchased from MilliporeSigma. Ac_4_Res-Glc **1a**, Res-DAT **2**, and Res-FucNAc **3** were synthesized via standard organic chemistry techniques and characterized by ^1^H/^13^C NMR and mass spectrometry. Compounds were purified using flash silica gel chromatography.

### Resorufin Glycoside Activation by β-Glucosidase

Samples were arrayed in triplicate in a black 96-wellplate (ThermoFisher Scientific) for fluorescence measurements. Background fluorescence was measured by incubating 200 μM Res-Glc **1**, Res-DAT **2**, or Res-FucNAc **3** (from 2 mM DMSO stock solutions) in McIlvaine lysis buffer (0.16 M sodium phosphate, 0.07 M citric acid, pH 7). Experimental samples contained 200 μM resorufin glycosides in McIlvaine lysis buffer containing β-glucosidase (MilliporeSigma, 2 μM). Following 30 min of incubation at room temperature, samples were analyzed using a fluorescent plate reader (Promega GloMax Discover) with filter selection at wavelengths (λ_ex_ 520 nm, λ_em_ 580–640 nm) corresponding to resorufin’s excitation and emission maxima (λ_ex_ 535 nm, λ_em_ 585 nm).

### Cell Culture

*B. fragilis* and *B. thetaiotaomicron* were grown at 37 °C on brain-heart infusion plates for 24 h under anaerobic conditions generated by an Oxoid AnaeroGen sachet in an airtight container. *H. pylori* cells were grown at 37 °C under microaerophilic conditions (14% CO_2_) in Brucella broth. AGS cells were grown in Ham’s F12 Glutamax medium, supplemented with 10% fetal bovine serum, in 5% CO_2_ at 37 °C. Cells were harvested and rinsed three times with phosphate-buffered saline (PBS) prior to resuspension in PBS at an optical density at 600 nm (OD_600_) of 1.0 for bacterial cells or 1 × 10^6^ cells/mL for AGS cells. The PBS was used at a pH favorable for each cell type (pH 5 for *H. pylori* and pH 7 for other cells).

### Cell Lysis

After harvesting and then washing cells with PBS three times, cell pellets were resuspended in McIlvaine lysis buffer (pH 5 for *H. pylori* and pH 7 for *B. fragilis*, *B. thetaiotaomicron*, and AGS cells) containing 1X protease inhibitor cocktail (MilliporeSigma). To ensure cell lysis, samples were incubated at room temperature in lysis buffer for 10 min and then subjected to three rounds of freeze–thaw (−20 °C for 30 min and at 35 °C for 10 min). Bacterial samples were further lysed using probe sonication (Branson 250–450 Sonifier Analog Cell Disruptor, 2 min at 3.5 output and 25% duty). Lysates were centrifuged at 10,000*g* in a microcentrifuge for 10 min, and clarified lysate was separated from the pellet for use in resorufin activation assays.

### Concentration-Dependent Fluorogenic Glycoside Activation by *B. fragilis* Lysate over Time

To probe concentration-dependent fluorogenic glycoside activation by *B. fragilis* lysate over time, *B. fragilis* lysate was incubated with resorufin glycosides Res-Glc **1**, Res-DAT **2**, or Res-FucNAc **3** (0–200 μM), and fluorescence was monitored using a fluorescent plate reader (Promega GloMax Discover) over 60 min at room temperature. To capture background hydrolysis in the absence of *B. fragilis* lysate, McIlvaine lysis buffer containing 200 μM Res-Glc **1**, Res-DAT **2**, or Res-FucNAc **3** was incubated over 60 min at room temperature.

### Monitoring Fluorogenic Glycoside Activation by *B. fragilis* Live Cells over Time

To probe the dynamics of fluorogenic glycoside activation by *B. fragilis* over time, *B. fragilis* cells were incubated with 200 μM Res-Glc **1**, Ac_4_Res-Glc **1a**, Res-DAT **2**, or Res-FucNAc **3** (0–200 μM), and fluorescence was monitored using a fluorescent plate reader (Promega GloMax Discover) over 4 h at room temperature. To capture background hydrolysis in the absence of *B. fragilis* cells, PBS containing 200 μM Res-Glc **1**, Ac_4_Res-Glc **1a**, Res-DAT **2**, or Res-FucNAc **3** was incubated at room temperature and monitored in parallel to cell samples over 4 h.

### Monitoring Resorufin Glycoside Activation by Cells

Samples were arrayed in triplicate in a black 96-well plate (ThermoFisher) for fluorescence measurements. Background fluorescence was measured by incubating 200 μM Res-Glc **1**, Res-DAT **2**, or Res-FucNAc **3** (from 2 mM DMSO stock solutions) in PBS (pH 5 for *H. pylori* and pH 7 for other cells) for 60 min. Experimental samples contained 200 μM resorufin glycosides with *B. fragilis*, *B. thetaiotaomicron*, *H. pylori*, or AGS cells in PBS adjusted to the optimal pH, as indicated. Following incubation at room temperature, fluorescence was quantified using a fluorescent plate reader (Promega GloMax Discover) as described.

### In-Gel Fluorescence Assay to Monitor Protein-Mediated Activation of Resorufin Glycosides

Protein concentrations of clarified lysates from *B. fragilis* and β-glucosidase from MilliporeSigma were determined via the Bio-Rad DC Protein Assay (Bio-Rad, Hercules, CA), and samples were diluted in McIlvaine lysis buffer to a standardized concentration of 3.0 mg/mL. A solution of bovine serum albumin (BSA) (3.0 mg/mL in McIlvaine lysis buffer) was prepared for analysis. Standardized samples were diluted 1:1 with 2X sample buffer (62.5 mM Tris–HCl, pH 6.8, 25% glycerol, 1% bromophenol blue). Samples (15 μg) were loaded onto four polyacrylamide gels (12% Tris-glycine protein gel with a 4% stacking layer, Bio-Rad, Hercules, CA) for Native-PAGE electrophoresis. Gels were electrophoresed at 100 V for 5 min followed by 200 V for 40 min in 1X native running buffer (H_2_O, 25 mM Tris, 192 mM glycine, pH 8.3) in a Mini PROTEAN Tetra Cell (Bio-Rad, Hercules, CA). One electrophoresed gel was incubated in Coomassie stain (45% deionized water, 45% methanol, 10% acetic acid, 0.25% Coomassie brilliant blue) at room temperature for 25 min prior to destaining with Coomassie destaining buffer (50% deionized water, 40% methanol, 10% acetic acid) until protein bands were visible. The remaining three electrophoresed gels were incubated with 2 μM Res-Glc **1**, Res-DAT **2**, or Res-FucNAc **3** (from 2 mM DMSO stock solutions) in PBS (pH 7) for 15 min at room temperature. Following incubation, in-gel fluorescence was visualized by G-Box Chemiluminescent Imager (Syngene, Frederick, MD) using the Alexa Fluor 568 filter.

### In-Gel Digestion and Liquid Chromatography–Mass Spectrometry Analysis

The electrophoresed *B. fragilis* protein band that exhibited robust in-gel fluorescence following incubation with Res-Glc 1 (Figure 3A, asterisk) was excised, diced, prepared, and subjected to digestion with sequencing-grade trypsin (Promega). Following extraction of peptides from digested gel pieces and reconstitution in water, LC–MS/MS analysis was performed to identify peptide sequences present. Proteins corresponding to unique peptides were identified by analyzing a *B. fragilis* database and a database for likely contaminant proteins (e.g., keratin). Top protein hits corresponding to unique peptides identified and percent amino acid coverage are available in Supporting Information. Two independent replicates were performed and led to the identification of peptide fragments corresponding to the *B. fragilis* β-glucosidase BglX both times.

## Supplementary Material





## Notes

Excel spreadsheet with mass spectrometry data (XLSX)
Full experimental details, compound characterization data, high-performance liquid chromatography analyses, cell lysate activity data, and in-gel fluorescence data (PDF)

## Abbreviations

GH: glycoside hydrolase
CAZy: carbohydrate-active enzymes database
GlcNAc: *N*-acetylglucosamine
FRET: Forster resonance energy transfer
Res-Glc: resorufin-β-d-glucopyranoside
d-DATDG: d-2,4-diacetamido-2,4,6-trideoxy galactose
d-FucNAc: *N*-acetyl d-fucosamine
Res-DAT: resorufin-β-d-DATDG
Res-FucNAc: resorufin-β-d-FucNAc
NIS: *N*-iodosuccinimide
TfOH: triflic acid
TMSOTf: trimethylsilyl trifluoromethanesulfonate
AgOTf: silver trifluoromethanesulfonate
TLC: thin-layer chromatography
NMR: nuclear magnetic resonance
SBn: thiobenzyl
NBS: *N*-bromosuccinimide
THF: tetrahydrofuran
H_2_O: water
CCl_3_CN: trichloroacetonitrile
CH_2_Cl_2_: methylene chloride
NHAc: *N*-acetyl
AcOH: acetic acid
K_2_CO_3_: potassium carbonate
*B. fragilis*: *Bacteroides fragilis*
*B. theta*: *Bacteroides thetaiotaomicron*
*H. pylori*: *Helicobacter pylori*
AGS: gastric adenocarcinoma
ATCC: American Type Culture Collection
OD_600_: optical density at 600 nm
PBS: phosphate-buffered saline
EDTA: ethylenediaminetetraacetic acid
LC-MS/MS: liquid chromatography–tandem mass spectrometry
