# Supplementary material for: Development of Fluorogenic Probes to Detect Glycan-Degrading Enzymes in Bacteria
Source: ACS Infect Dis. 2026 Jul 20;12(8):2778–87. doi: 10.1021/acsinfecdis.6c00347 (PMC13488444; doi:10.1021/acsinfecdis.6c00347)
Supplement: Supplementary file 1 [file id6c00347_si_002.pdf]

Supporting Information

Development of fluorogenic probes to detect glycan-degrading enzymes in bacteria

Esteban Tarazona Guzman,[1#] Ankita Paul,[2#] Karen D. Moulton,[1] Daniel E. Calles-Garcia,[1] Maren Cooper,[1] Lucas DiCerbo,[1] Soumyakanta Maji,[2] Elizabeth A. Stemmler,[1] Suvarn S. Kulkarni,[2*] and Danielle H. Dube[1*]

[1]Department of Chemistry & Biochemistry, Bowdoin College,
2 Polar Loop, Brunswick, ME 04011, USA.
[2]Department of Chemistry, Indian Institute of Technology Bombay,
Powai, Mumbai 400-076, India.
*Corresponding authors: ddube@bowdoin.edu, suvarn@chem.iitb.ac.in

[#]These authors contributed equally to this work.

Table of Contents

Chemistry ***2***

General 2

Compound 7 2

Compound 2 3

Compound 9 4

Compound 3 5

Compound 1a 6

Biology ***7***

Methods 7

High performance liquid chromatography analyses 7

Cell preparation and lysis 8

Monitoring resorufin glycoside activation by cell lysates 8

In gel fluorescence assay to monitor protein-mediated activation of resorufin glycosides 8

Gel digestion to identify a glycosidase corresponding to Res-Glc 1 activation 9

LC-MS/MS analysis of gel digests 10

Database searching 10

Database search results 11

Supplemental Figures ***12***

Chemistry

General

All reactions were conducted under the dry nitrogen atmosphere. Solvents (CH_2Cl_2 >99%, THF 99.5%, acetonitrile 99.8%, DMF 99.5%) were purchased in capped bottles and dried under sodium or CaH_2. All other solvents and reagents were used without further purification. Glassware was oven dried before use. Reactions under heating condition were performed using paraffin oil bath. TLC was performed on pre-coated Aluminium plates of Silica Gel 60 F254 (0.25 mm, E. Merck). Developed TLC plates were visualized under a short-wave UV lamp and by heating plates that were dipped in ammonium molybdate/cerium (IV) sulphate solution. Silica gel column chromatography was performed using Silica Gel (100-200 mesh, as well as 230-400 mesh) and employed a solvent polarity correlated with TLC mobility. We used 3 Angstrom powdered molecular sieves in our study. The powdered molecular sieves were weighed in a dried pear-shaped flask and activated by periodic heating of flask by using flame over a period of 15 minutes. NMR experiments were conducted on 500 and 400 MHz instrument using $CDCl_3$ (D, 99.8%), CD_3OD (D, 99.8%) or D_2O (D, 99.9%) as solvents. Chemical shifts are relative to the deuterated solvent peaks and are in parts per million (ppm). In the ^{1}H NMR spectrum, data are reported as follows: chemical shift (δ ppm), multiplicity (s: singlet, d: doublet, dd: doublet of doublet, t: triplet, q: quartet, m: multiplet, bs: broad singlet and ABq: AB quartet), coupling constant (J in Hz), integration and respective assigned proton(s). Mass spectra were acquired in the ESI-TOF mode. Specific rotation experiments were measured at 589 nm (Na) and 25 °C. IR spectra were recorded on an FT-IR spectrometer.

Compound 7

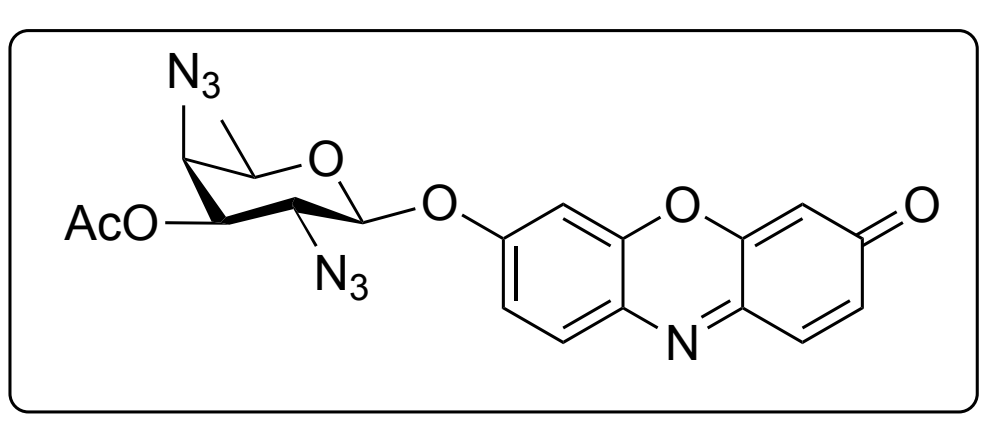

Compound **4** (0.044 g, 0.126 mmol) was azeotroped with dry toluene and kept in vacuum for overnight. Compound **4** and activated MS (0.200 g) was dissolved in dry CH_2Cl_2 (10 ml). At 0 °C,

bromine (0.01 ml, 0.277 mmol) was added dropwise. After 30 mins, TLC shows consumption of starting material and formation of non-polar bromo compound. Reaction mixture was azeotroped with dry toluene, concentrated under N_2 atm via rotory evaporator, and kept in vacuum for 30 mins before the next step. Next, resorufin sodium salt (0.023 g, 0.097 mmol), Ag_2CO_3 (0.069 g, 0.252 mmol) along with activated molecular sieves was dissolved in dry DMF (2 ml). Then, the bromo compound was cannulated to this solution and stirred at RT for 8 h. Reaction mixture was filtered through celite, concentrated and purified by column chromatography to get compound **7** (0.023 g, 52% over two steps, β-isomer).

$[\alpha]_D^{25}$ +12.60 (c = 0.2, $CHCl_3$).

IR (cm^{-1}, $CHCl_3$) ν 3891, 2110, 1742, 1689, 1547, 549, 486.

^{1}H NMR (400 MHz, $CDCl_3$) δ 7.76 (d, 1H, J = 8 Hz), 7.45 (d, 1H, J = 10 Hz), 7.07 (dd, 1H, J = 8.8 Hz, 2.4 Hz), 6.97 (d, 1H, J = 2.8 Hz), 6.87 (dd, 1H, J = 9.6 Hz, 2 Hz), 6.36 (d, 1H, J = 2 Hz), 4.99-4.97 (m, 2H, H-1, H-3), 4.08 (dd, 1H, J = 10.8 Hz, 8 Hz, H-2), 3.95-3.93 (m, 2H, H-4, H-5), 2.25 (s, 3H, OAc), 1.46 (d, 3H, J = 6.4 Hz, H-6).

^{13}C{1H} NMR NMR (100 MHz, $CDCl_3$) δ 186.36, 170.07, 159.79, 149.57, 145.14, 134.81, 134.71, 131.71, 114.96, 107.04, 103.51, 99.93, 73.30, 70.00, 64.40, 60.25, 29.71, 20.56, 17.38.

HR-ESI-MS (*m/z*): $[M + H]^+$ calcd. for $C_{20}H_{18}N_7O_6$, 452.1345; found, 452.1344.

Compound 2

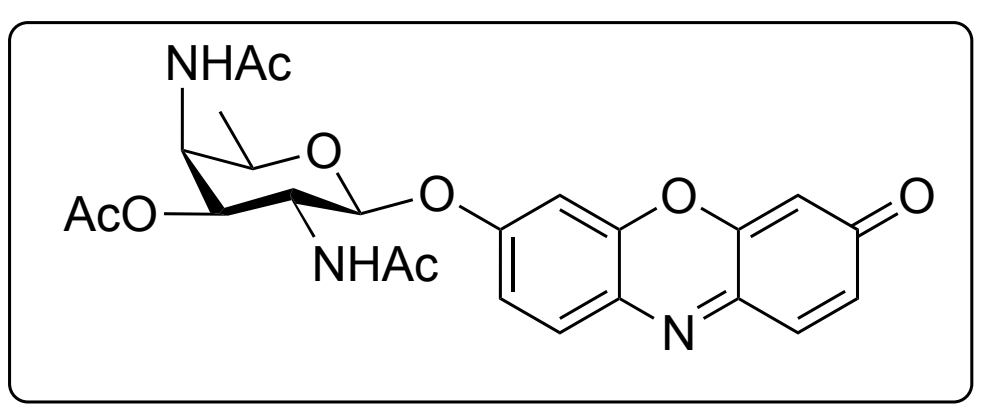

Compound **7** (0.020 g, 0.044 mmol) was taken in THF (2 ml) and PMe_3 in 1M toluene (0.221 mmol, 0.2 ml) was added to it. After, stirring for 30 mins, a fluorescent polar spot is observed. Reaction mixture was concentrated, azeotroped with toluene and kept in vacumn. For next step, crude reaction mixture was taken in THF (2 ml), Ac_2O (0.02 ml, 0.264 mmol) and Et_3N (0.02 ml) were added sequentially and stirred at RT for 1 h. After completion of reaction, reaction mixture was concentrated in vacuo and purified through column chromatography (10% Methanol-EA eluted the product) to give compound **2** (0.015 g, 68%).

[*α*]$_D^{25}$ +7.5 (c = 0.15, $CHCl_3$).

IR (cm^{-1}, $CHCl_3$) *ν* 3542, 2615, 1674, 1554, 549, 486.

^{1}H NMR (400 MHz, $CDCl_3$) *δ* 7.70 (d, 1H, *J* = 8.8 Hz), 7.40 (d, 1H, *J* = 9.6 Hz), 7.00 (dd, 1H, *J* = 8.8 Hz, 2.8 Hz), 6.91 (d, 1H, *J* = 2.4 Hz), 6.84 (dd, 1H, *J* = 9.6 Hz, 2 Hz), 6.32 (d, 1H, *J* = 2 Hz), 6.06 (d, 1H, *J* = 9.6 Hz, NH), 5.87 (d, 1H, *J* = 8.8 Hz, NH),), 5.25-5.19 (m, 2H, H-1, H-3), 4.58 (dd, 1H, *J* = 9.6 Hz, 3.2 Hz, H-4), 4.31-4.34 (m, 1H, H-2), 4.06-4.04 (m, 1H, H-5), 2.25 (s, 3H, OAc), 2.09 (s, 3H, NHAc), 2.03(s, 3H, NHAc), 1.33 (d, 3H, *J* = 6.4 Hz, H6)).

^{13}C{1H} NMR (100 MHz, $CDCl_3$) *δ* 186.31, 171.04, 170.77, 170.61, 160.31, 149.47, 146.68, 145.11, 134.78, 124.58, 131.69, 129.44, 114.67, 107.04, 103.24, 99.48, 70.84, 70.50, 51.01, 50.19, 23.43, 23.24, 20.84, 16.69.

HR-ESI-MS (*m/z*): $[M + H]^+$ calcd. for $C_{21}H_{29}N_6O_9$, 509.2005; found, 509.2004.

Compound 9

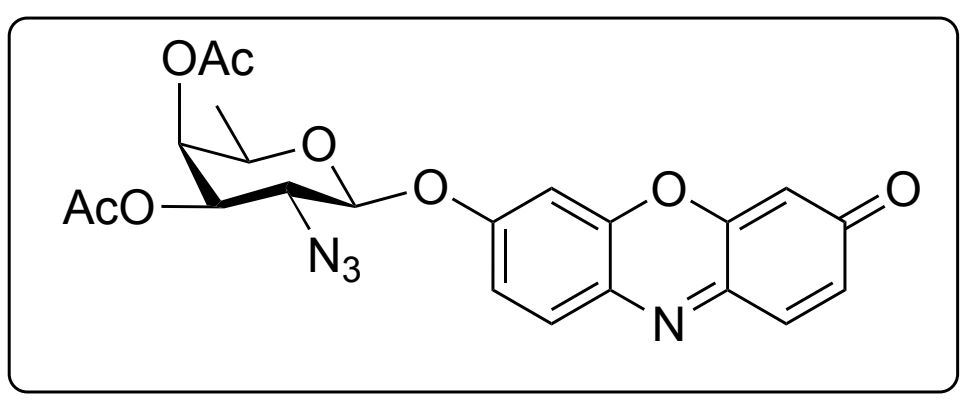

Compound **8** (0.124 g, 0.339 mmol) was azeotroped with dry toluene and kept in vacuum for overnight. Compound **8** and activated MS (0.200 g) was dissolved in dry CH_2Cl_2 (10 ml). At 0 °C, bromine (0.04 ml, 0.746 mmol) was added dropwise. After 30 mins, TLC shows consumption of starting material and formation of non-polar bromo compound. Reaction mixture was azeotroped with dry toluene, concentrated under N_2 atm in rotor evaporator and kept in vacuum for 30 mins before the next step. Next, resorufin sodium salt (0.053 g, 0.226 mmol), Ag_2CO_3 (0.186 g, 0.678 mmol) along with activated molecular sieves was dissolved in dry DMF (2 ml). Then, the bromo compound was cannulated to this solution and stirred at RT for 8 h. Reaction mixture was filtered through celite, concentrated and purified by column chromatography to get compound **9** (0.037 g, 55% over two steps).

[*α*]$_D^{25}$ +17.2 (c = 0.1, $CHCl_3$).

IR (cm^{-1}, $CHCl_3$) ν 3487, 2109, 1667, 1547, 549, 486.

^{1}H NMR (500 MHz, $CDCl_3$) δ 7.78 (d, 1H, J = 8.8 Hz), 7.46 (d, 1H, J = 9.6 Hz), 7.29 (s, 1H), 7.10 (d, 1H, J = 2.4 Hz), 6.88 (d, 1H, J = 2 Hz), 6.37 (s, 1H), 5.32 (s, 1H, H-4), 5.06 (d, 1H, J = 7.5 Hz, H-1), 4.94 (d, 1H, J = 10 Hz, H-3), 4.04 (bs, 2H, H-5, H-2), 2.25 (s, 3H, OAc), 2.12 (s, 3H, OAc), 1.34 (d, 3H, J = 3.6 Hz, H-6).

^{13}C{1H} NMR (125 MHz, $CDCl_3$) δ 186.36, 170.34, 169.81, 159.81, 149.58, 146.93, 145.18, 134.81, 134.74, 131.75, 114.86, 107.07, 103.51, 100.02, 71.19, 70.06, 69.11, 60.35, 20.65, 20.63, 16.24.

HR-ESI-MS (*m/z*): $[M + Na]^+$ calcd. for $C_{22}H_{20}N_4NaO_8$, 491.1201; found, 491.1201.

Compound 3

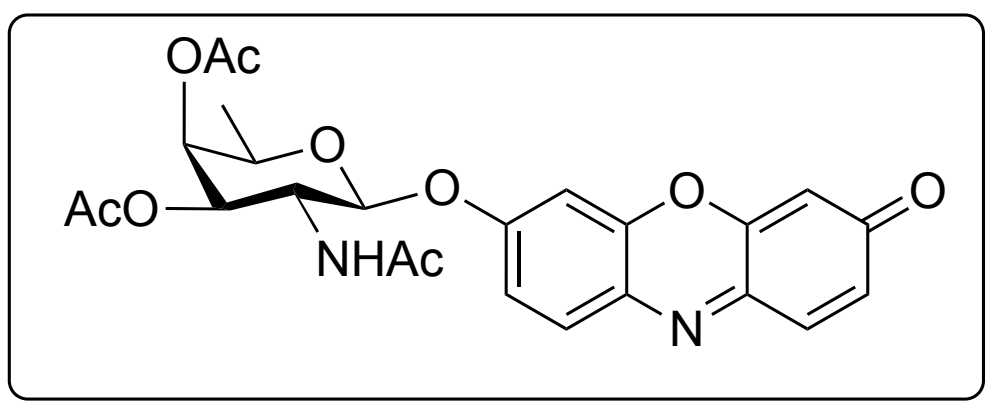

Compound **9** (0.020 g, 0.043 mmol) was taken in THF (2 ml) and PMe_3 in 1M toluene (0.102 mmol, 0.1 ml) was added to it. After, stirring for 30 mins, a fluorescent polar spot was observed. Reaction mixture was concentrated, azeotroped with toluene and kept in vacumn. Next, crude reaction mixture was taken in THF (2 ml), Ac_2O (0.01 ml, 0.128 mmol) and Et_3N (0.01 ml) were added sequentially and stirred at RT for 1 h. After completion of reaction, reaction mixture was concentrated in vacuo and purified through column chromatography (10% Methanol-EA eluted the product) to give compound **3** (0.014 g, 68%).

$[\alpha]_D^{25}$ +8.3 (c = 0.18, $CHCl_3$).

IR (cm^{-1}, $CHCl_3$) ν 3581, 1742, 1689, 1315, 509.

^{1}H NMR (500 MHz, $CDCl_3$) δ 7.73 (d, 1H, J = 8.8 Hz), 7.40 (d, 1H, J = 9.6 Hz), 7.03 (dd, 1H, J = 8.8 Hz, 2.8 Hz), 6.95 (d, 1H, J = 2.4 Hz, NH), 6.84 (dd, 1H, J = 9.6 Hz, 2 Hz, NH), 6.31 (d, 1H, J = 2 Hz), 5.76 (d, 1H, J = 5.0 Hz, H-4), 5.48-5.43 (m, 2H, H-3, H-1), 4.31-4.30 (m, 1H, H-2),

4.09-4.08 (m, 1H, H-5), 2.24 (s, 3H, OAc), 2.08 (s, 3H, OAc), 2.01 (s, 3H, NHAc), 1.33 (d, 3H, J = 8 Hz, H-6).

^{13}C{1H} NMR (125 MHz, CDCl$_3$) δ 186.31, 171.04, 170.71, 170.61, 160.31, 149.47, 146.68, 145.11, 134.78, 134.58, 131.69, 129.44, 114.67, 107.04, 103.24, 99.48, 70.84, 70.50, 51.01, 50.19, 23.43, 23.24, 20.84, 16.69.

HR-ESI-MS (*m/z*): $[M + H]^+$ calcd. for $C_{24}H_{25}N_2O_9$, 484.1508; found, 484.1507.

Compound 1a

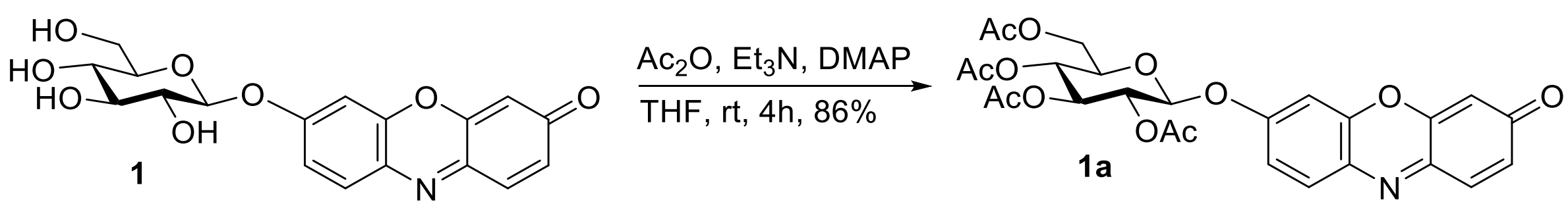

Compound **1** (30 mg, 0.08 mmol) was dissolved in THF (2 mL). To the clear solution, Ac_2O (0.04 mL, 0.40 mmol), Et_3N (0.05 mL) and DMAP (2.0 mg, 0.016 mmol) were added sequentially at room temperature, mixture was allowed to stir at room temperature for 4 h. After completion of reaction, solvents were removed in *vacuo* and the crude product was purified by column chromatography over silica gel (60% ethyl acetate: petroleum ether) to furnish desired substrate **1a** as red colour sticky liquid (43 mg, 86%).

$[\alpha]_D^{25}$ +9.16 (c = 0.25, CHCl$_3$).

IR (cm^{-1}, CHCl$_3$) ν 3021, 1746, 1669, 1432, 1287, 845.

^{1}H NMR (400 MHz, CDCl$_3$) δ 7.70 (d, J = 8.8 Hz, 1H, ArH), 7.40 (d, J = 9.8 Hz, 1H, ArH), 6.98-6.93 (m , 2H) 6.82 (dd, J = 9.8, 2.0 Hz, 1H, ArH), 6.27 (d, J = 2.0 Hz, 1H, ArH), 5.32 (t, J = 9.2 Hz, 1H, H-2), 5.29 (t, J = 9.1 Hz, 1H, H-3), 5.20 (d, J = 7.4 Hz, 1H, H-1), 5.15 (d, J = 9.4 Hz, 1H, H-4), 4.26 (dd, J = 12.3, 5.8 Hz, 1H, H-6a), 4.20 (dd, J = 12.3, 2.3 Hz, 1H, H-6b), 3.98-3.93 (m, 1H, H-5), 2.09 (s, 3H, $COCH_3$), 2.06 (s, 3H, $COCH_3$), 2.05 (s, 3H, $COCH_3$), 2.03 (s, 3H, $COCH_3$)

^{13}C{1H} NMR (100 MHz, CDCl$_3$) δ 186.3, 170.4, 170.2, 169.4, 169.2, 159.8, 149.5, 146.9, 145.1, 134.8, 134.7, 131.6, 129.6, 115.0, 107.0, 103.4, 98.3, 72.5, 70.9, 68.1, 61.9, 20.7, 20.63, 20.59

HRMS (ESI-TOF) (m/z): $[M+Na]^+$ calcd. for $C_{26}H_{25}NO_{12}Na$ 566.1269; found, 566.1269.

Biology

Methods

All organic reagents were obtained from MilliporeSigma. *H. pylori* strain G27 was obtained from Dr. Manuel Amieva (Stanford University). *B. fragilis* (ATCC 23745), *B. thetaiotaomicron* (ATCC 700349), and AGS cells (ATCC CRL-1739) were purchased from ATCC and cultured according to the manufacturer's instructions. Resorufin-β-D-glucopyranoside **1** and β-Glucosidase from almonds were purchased from MilliporeSigma.

High performance liquid chromatography analyses

Synthetic standards of 200 µM Res-Glc **1** and resorufin sodium salt (MilliporeSigma) were prepared in 95% 40 mM phosphate buffer (pH 6.8) and 5% acetonitrile. Resorufin-Glucose (200 µM) was incubated with *β*-glucosidase (MilliporeSigma, 10 µM) for 20 minutes at room temperature in 95% 40 mM phosphate buffer (pH 6.8) and 5% acetonitrile. Following incubation, samples were immediately filtered using a Pierce concentrator PES filter (Product Number: 88513, PES, 10K MWCO, 0.5mL, Thermo FisherScientific, Waltham, MA). Resorufin and Res-Glc **1** standards (200 µM) were also filtered alongside the experimental sample. Once filtered, all three samples were analyzed by HPLC. HPLC-grade 40 mM phosphate buffer was prepared by combining deionized water with appropriate proportions of sodium phosphate and sodium diphosphate, followed by pH adjustment and filtration using a MilliporeSigma™ classic glass filter holder kit, 47 mm (Product Number: XX1014730) with a 0.2 µm pore size nylon membrane filter (Product Number: GNWP02500). Chromatographic separation was performed using a Luna 3 µm C18(2) 100 Å, LC Column, 100 × 4.6 mm (Phenomenex, Torrance, CA). An isocratic method of 90% phosphate buffer and 10% acetonitrile at a flow rate of 1 mL/min was used for sample analysis. Material eluting from the column was detected using an Agilent 1100 series diode-array detector (DAD) measuring absorbance at 470 nm and 574 nm (Agilent Technologies, Santa Clara, CA).

Cell preparation and lysis

B. fragilis and *B. thetaiotaomicron* were grown at 37 ºC on brain-heart infusion plates for 24 hours under anaerobic conditions generated by an Oxoid AnaeroGen Sachet in an airtight container. *H. pylori* cells were grown at 37 ºC under microaerophilic conditions (14% CO_2) in Brucella Broth. AGS cells were grown in Ham's F12 Glutamax medium in 5% CO_2 at 37 ºC. Cells were harvested and rinsed three times with phosphate-buffered saline (PBS) prior to resuspension in PBS at an optical density 600 (OD_{600}) of 1.0 for bacterial cells or 1 x 10^6 cells/mL for AGS cells. Cells were pelleted by centrifugation and resuspended in McIlvaine lysis buffer (pH 5 for *H. pylori* and pH 7 for *B. fragilis, B. thetaiotaomicron*, and AGS cells) containing 1X protease inhibitor cocktail (MilliporeSigma). To ensure cell lysis, samples were incubated at room temperature in lysis buffer for 10 minutes, then subjected to three rounds of freeze-thaw (-20 ºC for 30 minutes and at 35 ºC for 10 minutes). Bacterial samples were further lysed using probe sonication (Branson 250 - 450 Sonifier Analog Cell Disruptor, 3.5 output control and 25% duty cycle, with 10 second pulses and 50 second rests during a 5-minute period). Lysates were centrifuged at 10,000 x *g* in a microcentrifuge for 10 minutes and clarified lysate was separated from the pellet for use in resorufin activation assays.

Monitoring resorufin glycoside activation by cell lysates

Samples were arrayed in triplicate in a black 96-well plate (ThermoFisher) for fluorescence measurements. Background fluorescence was measured by incubating 200 μM Res-Glc **1,** Res-DAT **2**, or Res-FucNAc **3** (from 2 mM DMSO stock solutions) in McIlvaine lysis buffer (pH 5 for *H. pylori* and pH 7 for other cells) for 30 minutes. Experimental samples contained 200 μM resorufin glycosides incubated with *B. fragilis* lysates, *B. thetaiotaomicron* lysates, *H. pylori* lysates, or AGS lysates in McIlvaine lysis buffer adjusted to the optimal pH, as indicated. Following incubation at room temperature, fluorescence was quantified using a fluorescent plate reader (Promega GloMax Discover) as described.

In gel fluorescence assay to monitor protein-mediated activation of resorufin glycosides

Protein concentrations of clarified lysates from *B. fragilis, H. pylori,* and β-glucosidase from MilliporeSigma were determined via the Bio-Rad DC Protein Assay (Bio-Rad, Hercules, CA), and

samples were diluted in McIlvaine lysis buffer to a standardized concentration of 3.0 mg/mL. A solution of bovine serum albumin (BSA) (3.0 mg/mL in McIlvaine lysis buffer) was prepared for analysis. Standardized samples were diluted 1:1 with 2X sample buffer (62.5 mM Tris-HCl, pH 6.8, 25% glycerol, 1% bromophenol blue). Samples (15 µg) were loaded onto four polyacrylamide gels (12% Tris-HCl protein gel with a 4% stacking layer, Bio-Rad, Hercules, CA) for Native-PAGE electrophoresis. Gels were electrophoresed at 100 V for 5 minutes followed by 200 V for 40 minutes in 1X native running buffer (H_2O, 25 mM Tris, 192 mM glycine, pH 8.3) in a Mini PROTEAN Tetra Cell (Bio-Rad, Hercules, CA). One electrophoresed gel was incubated in Coomassie stain (45% deionized water, 45% methanol, 10% acetic acid, 0.25% Coomassie brilliant blue) at room temperature for 25 minutes prior to destaining with Coomassie destaining buffer (50% deionized water, 40% methanol, 10% acetic acid) until protein bands were visible. The remaining three electrophoresed gels were incubated with 2 µM Res-Glc **1,** Res-DAT **2**, or Res-FucNAc **3** (from 2 mM DMSO stock solutions) in PBS (pH 7) for 15 minutes at room temperature. Following incubation, in-gel fluorescence was visualized by G-Box Chemiluminescent Imager (Syngene, Frederick, MD) using the Alexa Fluor 568 filter.

Gel digestion to identify a glycosidase corresponding to Res-Glc 1 activation

The electrophoresed *B. fragilis* protein band that exhibited robust in gel fluorescence following incubation with Res-Glc **1** (Fig. 3A, asterisk) was excised, diced, and subjected to three, ten-minute washes with 25 mM NH_4HCO_3 /50% ACN. Gel pieces were incubated with 10 mM dithiothreitol/25 mM NH_4HCO_3 for one hour at 56 °C, then the solution was removed and gel pieces were incubated for 45 minutes with 55 mM iodoacetamide/25 mM NH_4HCO_3 in the dark at room temperature. Gel pieces were then washed for ten minutes with 25 mM NH_4HCO_3, followed by two, five-minute washes with 25 mM NH_4HCO_3 /50% ACN. Gel pieces were then dried completely with a SpeedVac. Sequencing grade trypsin (Promega, 100 ng in 25 mM NH_4HCO_3) was added to the gel pieces and left at 4 °C for ten minutes. Additional 25 mM NH_4HCO_3 was added to fully cover the gel pieces, and samples were left to incubate at 37°C for twenty hours. Following trypsin incubation, aqueous digests were collected. Gel pieces were incubated for twenty minutes with 50% ACN/5% TFA, then sonicated for five minutes and the supernatant was combined with previous aqueous digests. This process was repeated once, then the combined

extracted digests were dried completely with a SpeedVac. Samples were reconstituted in LCMS water with 0.1% formic acid prior to LC-MS/MS analysis.

LC-MS/MS analysis of gel digests

LC-MS/MS analysis of tryptic peptides from gel bands was performed using a 6530 quadrupole time-of-flight (Q-TOF) mass analyzer (Agilent Technologies, Santa Clara CA) using Mass Hunter Data Acquisition V10.1 with chromatographic separation and nanoelectrospray ionization (nanoESI) performed using a 1260 Chip Cube system (Agilent Technologies) and a ProtID-chip with a 40 nL enrichment column and a 150-mm x 75 µm analytical column (Agilent Technologies). The enrichment and analytical columns were packed with 300 Å, 5 µm particles with Zorbax 300SB-C18 stationary phase. The mobile phases were 0.1% formic acid in H_2O (A) and 0.1% formic acid and 2% water in acetonitrile (B). Samples (4 µL) were loaded on the enrichment column using 98:2 (A:B) at 4 µL/min and the following gradient conditions: 98% A (1.0 min); 65% A (70 min); 30% A (75 min); 10% A (85 min). Mass spectra (MS and MS/MS) were collected in positive ion mode. Spectra were internally calibrated using dibutyl phthalate ($C_{16}H_{22}O_4$) and hexakis(1H, 1H, 4H-hexafluorobutyloxy)phosphazine (HP-1221; $C_{24}H_{18}O_6N_3P_3F_{36}$), continuously evaporated and detected as $[M+H]^+$. CID-MS/MS experiments were executed using data-dependent acquisition (DDA) with precursor ions (+2 or +3) preferenced and selected for collision-induced dissociation (CID) using nitrogen as the target gas.

Database searching

LC-MS/MS data (2 runs per sample) was subject to data extraction/peak picking/deisotoping and searching using the Spectrum Mill MS Proteomics Workbench (Version 7.11; Broad Institute), with experimental data searched against the *B. fragilis* protein database GCF_000025985.1_ASM2598v1_protein (downloaded 3/20/2026) and a contaminant database (Repository of Adventitious Proteins or cRAP) from the Global Proteome Machine (GPM; https://www.thegpm.org/crap/). Searches were conducted allowing up to 4 missed cleavages, carbamidomethylation of cysteine as a fixed modification, and methionine oxidation, pyroglutamic acid formation, deamination, and phosphorylation set as variable modifications. Precursor and fragment mass tolerances were set to 20 and 50 ppm, respectively. Peptide and protein identifications were accepted using an FDR of 1.2% across each run.

Database search results

Two independent replicates were performed with two LC-MS/MS analyses of each, which led to the identification of peptide fragments mapping onto *B. fragilis* β-glucosidase BglX proteins with the highest coverage (7.8 and 5.8%, respectively) associated with WP_010992000.1 (see SI MS final search results). The *B. fragilis* β-glucosidase BglX protein-associated tryptic peptides over eight amino acids in length identified by mass spectrometry analysis (IGQLNLPVTGEITTGQAK, VSEGSGEDPFLGALIAR, IYLQPNESR, and FTIAPEMLK) were subjected to NCBI Blast searches on 4/5/26 against the ClusteredNR database of all proteins, and the best hits for non-hypothetical proteins were glycosidases from the Bacteroides family. Searches of the latter two peptides yielded *B. fragilis* β-glucosidase BglX as the top hit.

Supplemental Figures

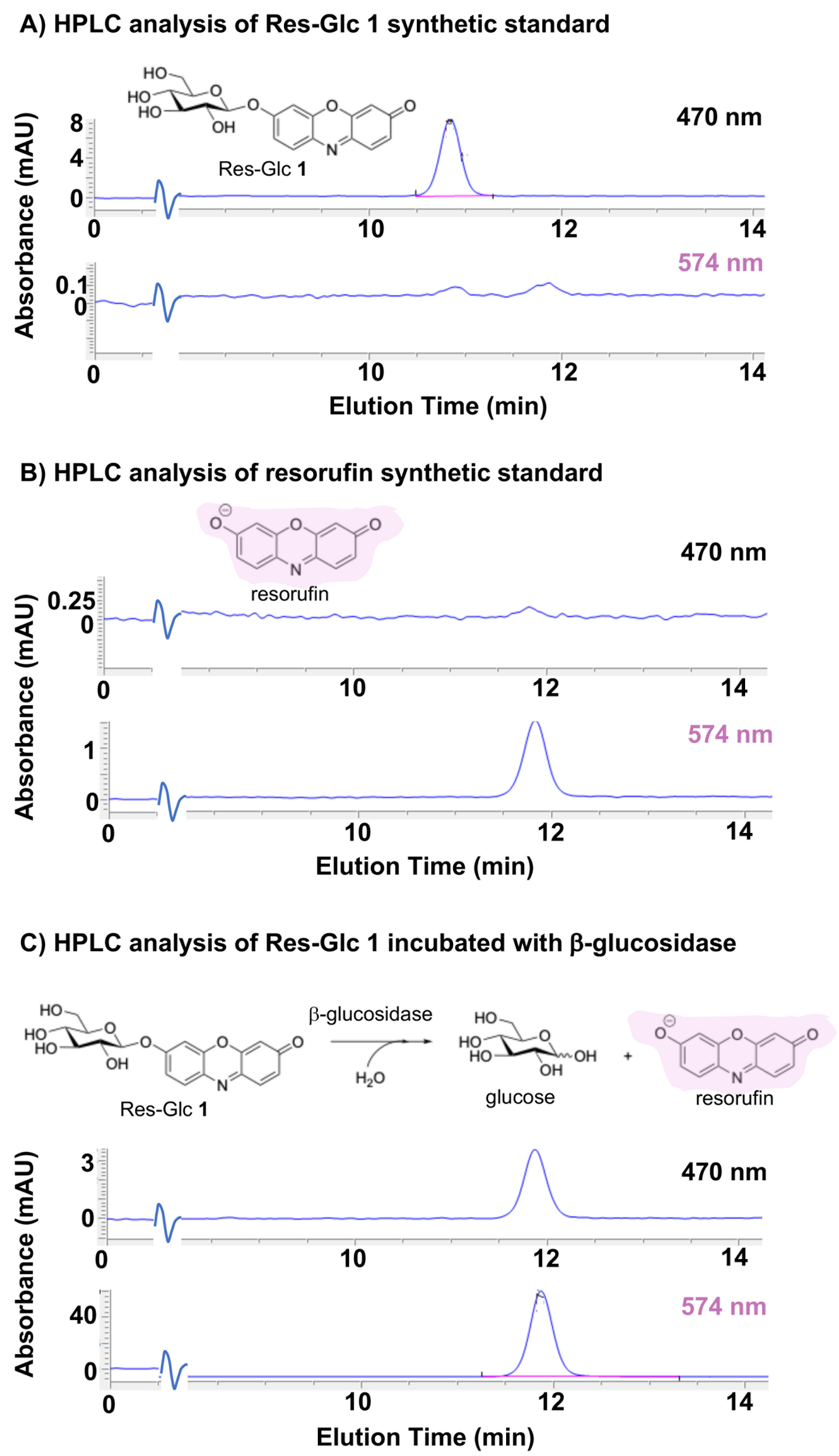

Figure S1. High performance liquid chromatography (HPLC) analysis confirms Res-Glc **1** treatment with *β*-glucosidase yields resorufin. (A) Res-Glc **1** synthetic standard, (B) resorufin synthetic standard, and (C) Res-Glc **1** after incubation with *β*-glucosidase were analyzed by HPLC on a C18 column. Absorbance was detected at 470 nm and 574 nm. Res-Glc **1** eluted at 10.8 minutes and resorufin eluted at 11.8 minutes. (C) The absence of the Res-Glc **1** peak at 10.8 minutes and the presence of the resorufin peak at 11.8 minutes following treatment of Res-Glc **1** with *β*-glucosidase confirms hydrolysis of the glycosidic bond of Res-Glc **1** to yield free resorufin.

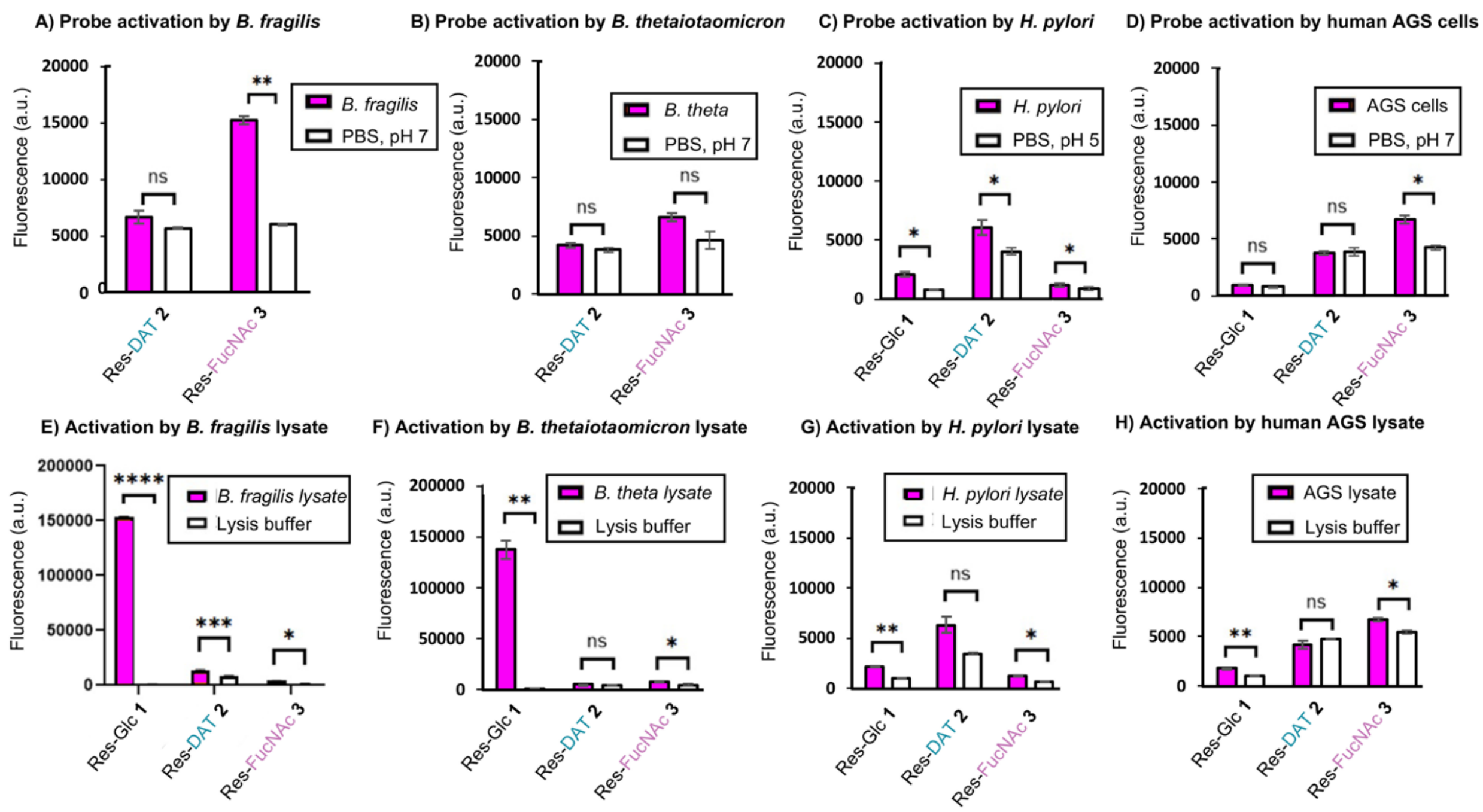

Figure S2. Fluorogenic resorufin glycosides are activated to varying extents by bacterial and mammalian cells (A-D) and lysates (E-H). (A-D) Data from Figure 2 (C-F) are shown with standardized y-axes for a direct comparison of relative activation of Res-DAT **2** and Res-FucNAc **3**. The indicated resorufin glycosides (200 μM) were incubated for 60 minutes in PBS with or without (A) *Bacteroides fragilis*, (B), *Bacteroides thetaiotaomicron (B. theta)*, (C) *Helicobacter pylori*, or (D) AGS cells. Following incubation, fluorescence at 585 nm was measured and compared to probes incubated in lysis buffer alone during the same interval. (E-H) The indicated resorufin glycosides (200 μM) were incubated for 30 minutes in lysis buffer with or without cell lysates from (E) *B. fragilis*, (F), *B. thetaiotaomicron (B. theta)*, (G) *H. pylori*, or (H) AGS cells. Following incubation, fluorescence at 585 nm was measured and compared to probes incubated in lysis buffer alone during the same interval. Error bars represent technical replicates from triplicate samples. Data are representative of replicate experiments (n = 3) that exhibited the same findings. Welch's t-test was used to determine statistical significance between paired control and experimental samples (*P < 0.01; ** P < 0.001; *** P < 0.0001; **** P < 0.00001; ns = non-significant).

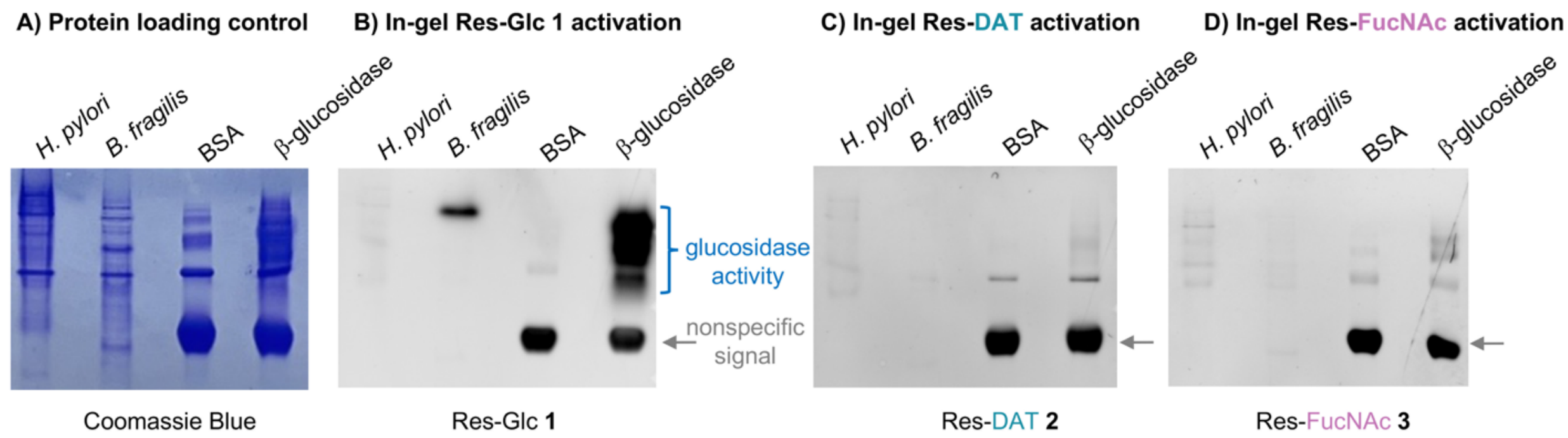

Figure S3. Resorufin-glucose activation by *B. fragilis* lysate can be pinpointed to one protein via in gel fluorescence analysis, while resorufin-glycoside activation by *H. pylori* lysate was negligible. *H. pylori* lysate, *B. fragilis* lysate, the negative control bovine serum albumin (BSA), and the positive control *β*-glucosidase were separated using native PAGE and (A) stained using Coomassie blue to visualize protein loading or incubated with 2 μM (B) Res-Glc **1**, (C) Res-DAT **2**, or (D) Res-FucNAc **3** for 15 minutes prior to measuring in gel fluorescence at 568 nm to visualize glycosidase activity. Bands corresponding to glucosidase activity and nonspecific signal are indicated. Data are representative of replicate experiments (n = 2).

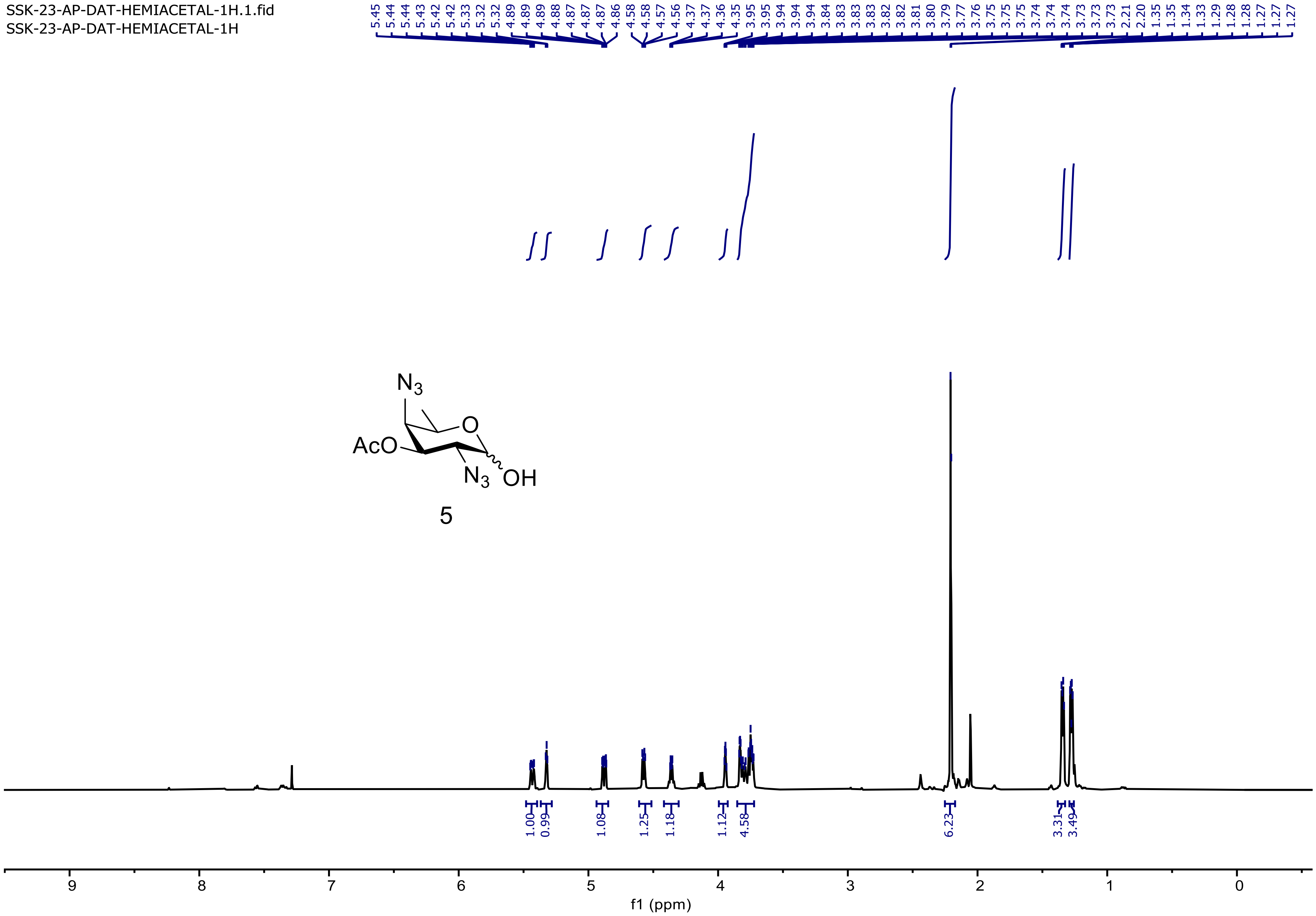
SSK-23-AP-DAT-HEMIACETAL-1H.1.fid
SSK-23-AP-DAT-HEMIACETAL-1H
5.45 5.44 5.44 5.43 5.42 5.42 5.33 5.32 5.32 4.89 4.89 4.88 4.87 4.87 4.87 4.86 4.58 4.58 4.57 4.56 4.37 4.37 4.36 4.35 3.95 3.95 3.94 3.94 3.84 3.83 3.83 3.83 3.82 3.82 3.81 3.80 3.79 3.77 3.76 3.75 3.75 3.75 3.74 3.74 3.74 3.73 3.73 3.73 2.21 2.20 1.35 1.35 1.34 1.33 1.29 1.28 1.27 1.27 1.27 1.27
N3
O
AcO
N3
OH
5
1.00 0.99 1.08 1.25 1.18 1.12 4.58 6.23 3.31 3.49
9 8 7 6 5 4 3 2 1 0
f1 (ppm)

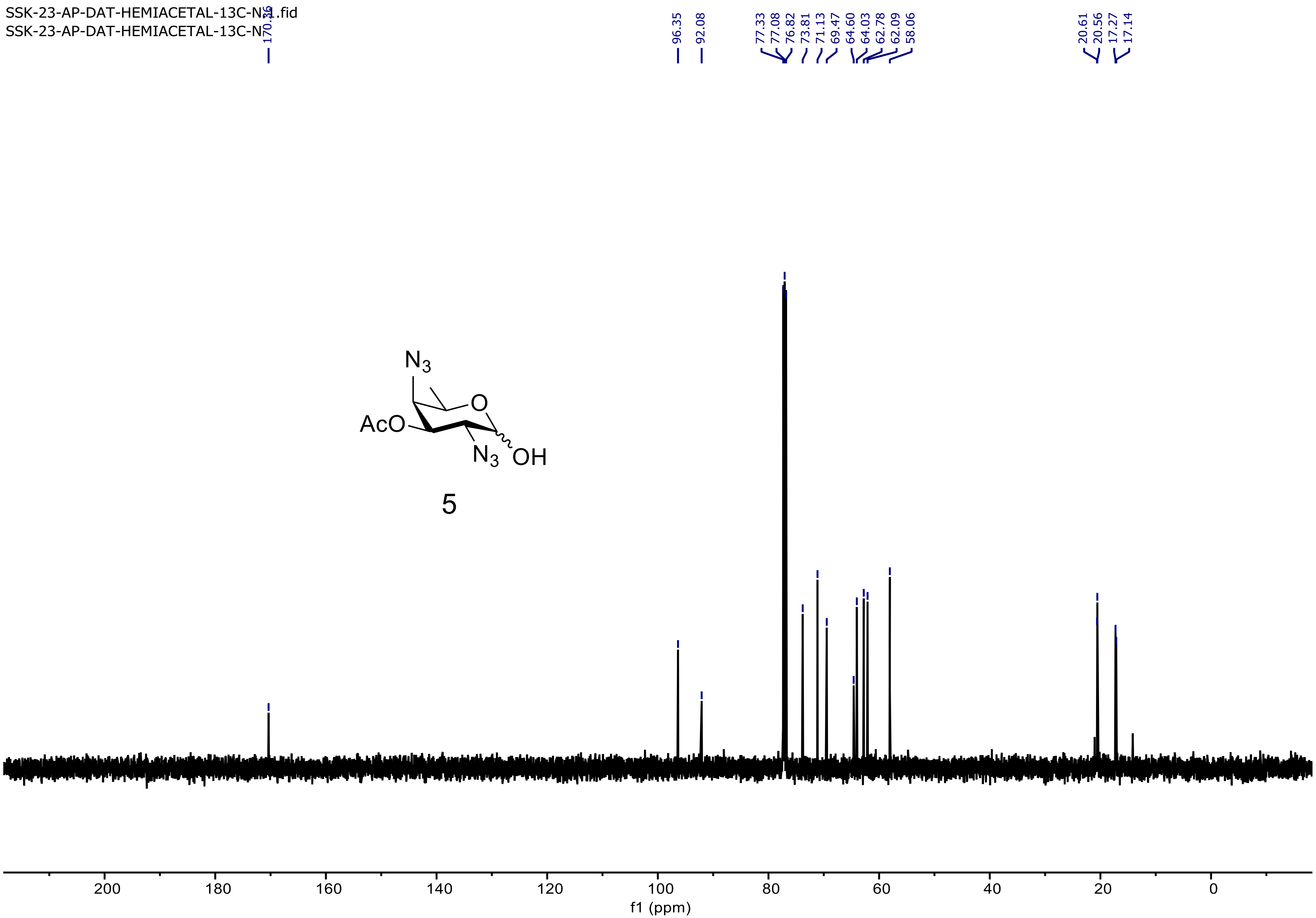
SSK-23-AP-DAT-HEMIACETAL-13C-N1.fid
SSK-23-AP-DAT-HEMIACETAL-13C-N
170.36
96.35
92.08
77.33
77.08
76.82
73.81
71.13
69.47
64.60
64.03
62.78
62.09
58.06
20.61
20.56
17.27
17.14
N3
O
AcO
N3
OH
5
200
180
160
140
120
100
80
60
40
20
0
f1 (ppm)

SSK-23-AP-990-1H

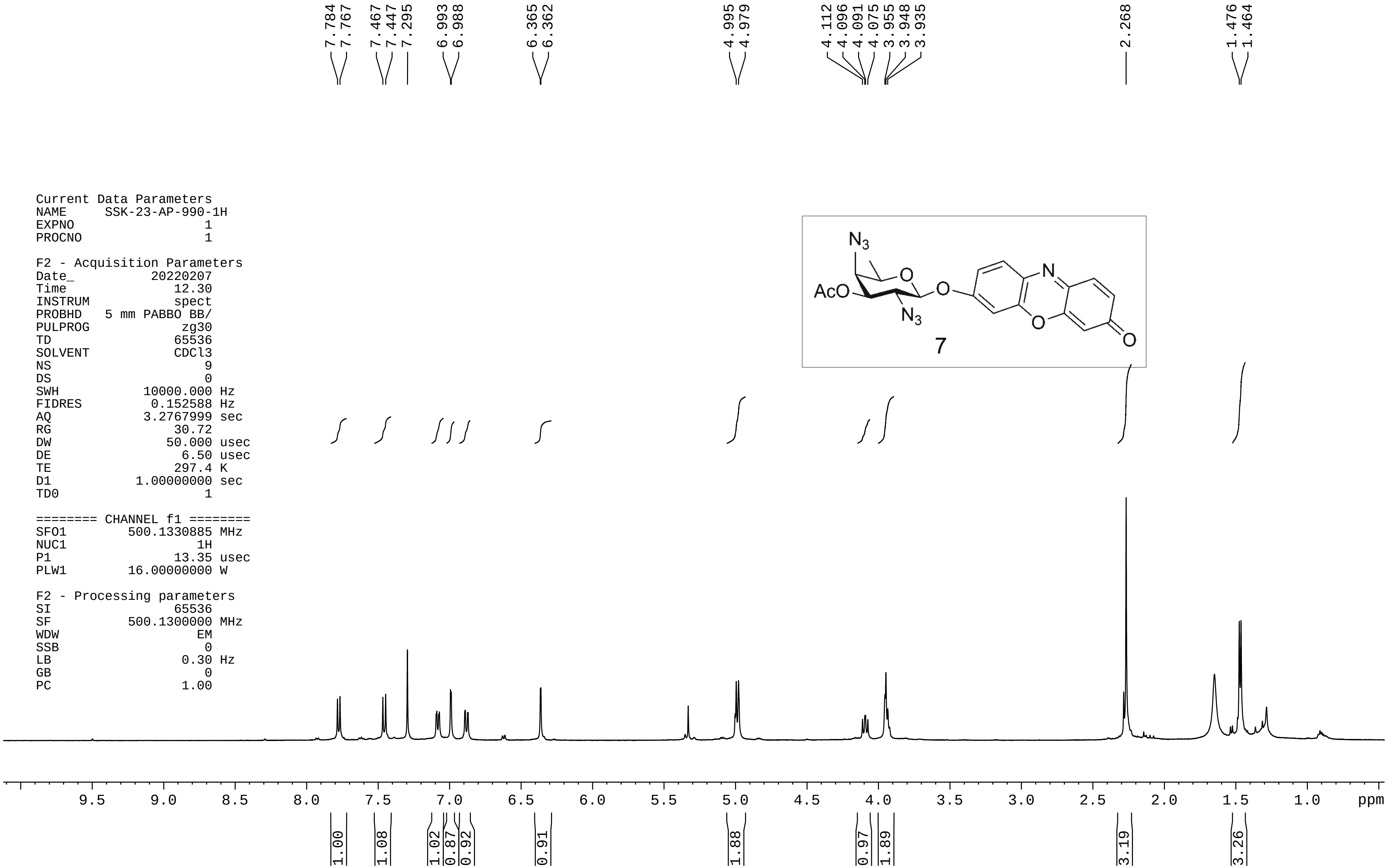

7

Current Data Parameters
NAME SSK-23-AP-990-1H
EXPNO 1
PROCNO 1

F2 - Acquisition Parameters
Date_ 20220207
Time 12.30
INSTRUM spect
PROBHD 5 mm PABBO BB/
PULPROG zg30
TD 65536
SOLVENT CDCl3
NS 9
DS 0
SWH 10000.000 Hz
FIDRES 0.152588 Hz
AQ 3.2767999 sec
RG 30.72
DW 50.000 usec
DE 6.50 usec
TE 297.4 K
D1 1.00000000 sec
TD0 1

======== CHANNEL f1 ========
SFO1 500.1330885 MHz
NUC1 1H
P1 13.35 usec
PLW1 16.00000000 W

F2 - Processing parameters
SI 65536
SF 500.1300000 MHz
WDW EM
SSB 0
LB 0.30 Hz
GB 0
PC 1.00

SSK-23-AP-990-13C-N

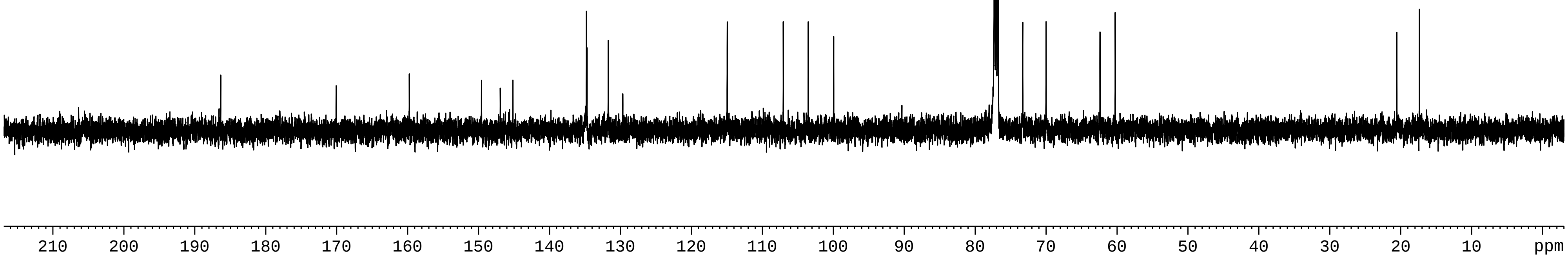


```
Current Data Parameters
NAME     SSK-23-AP-990-13C-N
EXPNO                      1
PROCNO                     1

F2 - Acquisition Parameters
Date_              20220207
Time                  12.12
INSTRUM               spect
PROBHD   5 mm PABBO BB/
PULPROG             zgpg30
TD                   65536
SOLVENT              CDCl3
NS                     370
DS                       0
SWH            29761.904 Hz
FIDRES          0.454131 Hz
AQ             1.1010048 sec
RG                  197.27
DW                  16.800 usec
DE                    6.50 usec
TE                   297.3 K
D1              1.00000000 sec
D11             0.03000000 sec
TD0                      1

======== CHANNEL f1 ========
SFO1         125.7703637 MHz
NUC1                   13C
P1                    8.90 usec
PLW1         103.00000000 W

======== CHANNEL f2 ========
SFO2         500.1320005 MHz
NUC2                    1H
CPDPRG[2           waltz16
PCPD2                80.00 usec
PLW2          16.00000000 W
PLW12          0.44556001 W
PLW13          0.22411001 W

F2 - Processing parameters
SI                   32768
SF             125.7577890 MHz
WDW                     EM
SSB                      0
LB                    1.00 Hz
GB                       0
PC                    1.40
```

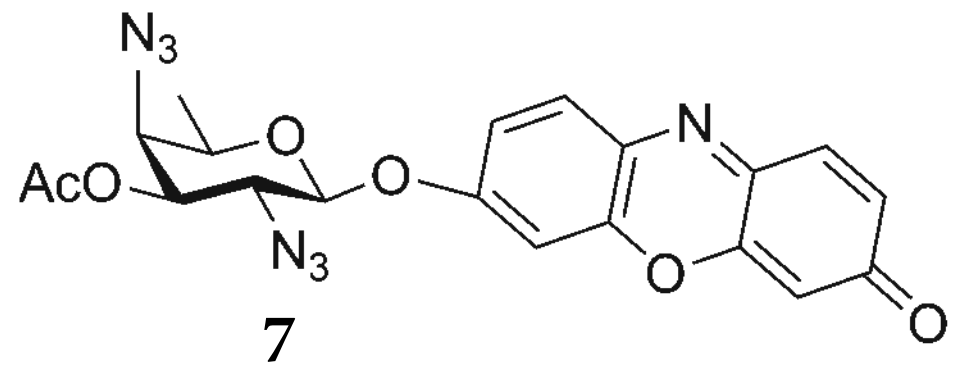

7

SSK-23-AP-990-COSY

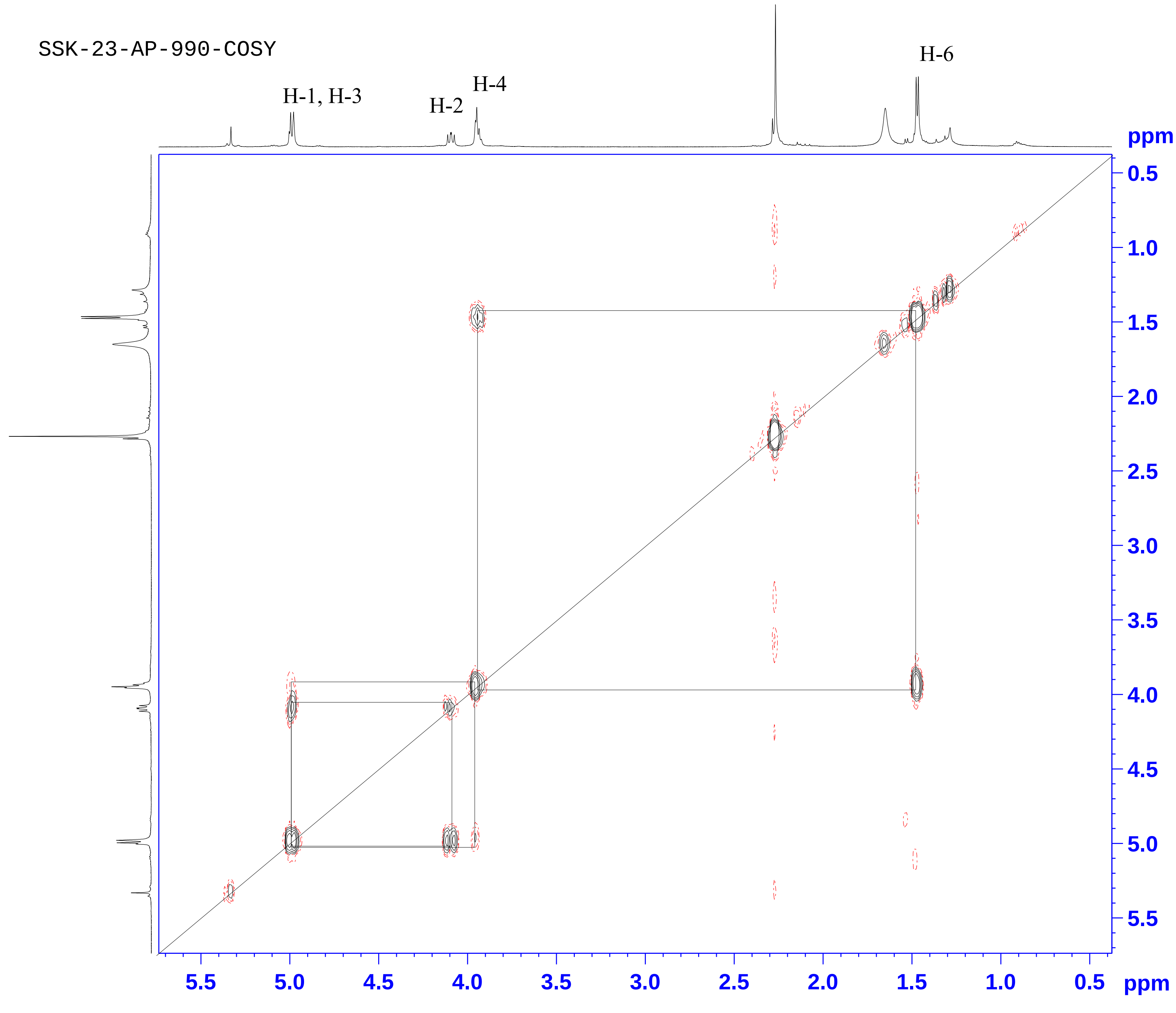

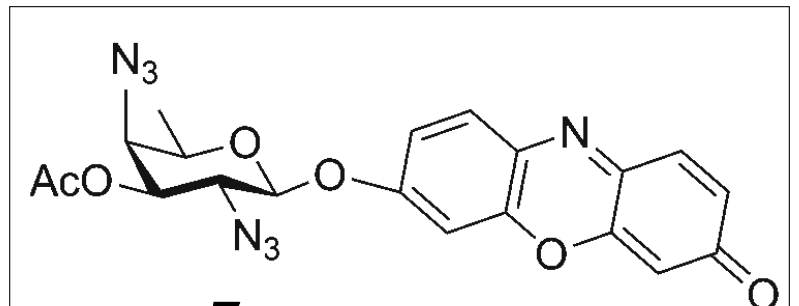

7

```
Current Data Parameters
NAME     SSK-23-AP-990-COSY
EXPNO                    1
PROCNO                   1

F2 - Acquisition Parameters
Date_             20220207
Time                 12.30
INSTRUM              spect
PROBHD   5 mm PABBO BB/
PULPROG       cosygpppqf
TD                    2048
SOLVENT              CDCl3
NS                       4
DS                       0
SWH               6684.492 Hz
FIDRES            3.263912 Hz
AQ               0.1531904 sec
RG                   61.42
DW                  74.800 usec
DE                    6.50 usec
TE                   297.4 K
D0              0.00000300 sec
D1              1.00000000 sec
D11             0.03000000 sec
D12             0.00002000 sec
D13             0.00000400 sec
D16             0.00020000 sec
IN0             0.00014960 sec

======== CHANNEL f1 ========
SFO1         500.1330069 MHz
NUC1                    1H
P0                   13.35 usec
P1                   13.35 usec
P17                5000.00 usec
PLW1           16.00000000 W
PLW10           3.16840005 W

====== GRADIENT CHANNEL =====
GPNAM[1]       SMSQ10.100
GPZ1                 10.00 %
P16                1000.00 usec

F1 - Acquisition parameters
TD                     128
SFO1               500.133 MHz
FIDRES          104.445190 Hz
SW                  13.365 ppm
FnMODE                  QF

F2 - Processing parameters
SI                    1024
SF             500.1300000 MHz
WDW                  QSINE
SSB                      0
LB                       0 Hz
GB                       0
PC                    1.40

F1 - Processing parameters
SI                    1024
MC2                     QF
SF             500.1300000 MHz
WDW                  QSINE
SSB                      0
LB                       0 Hz
GB                       0
```

SSK-23-AP-990-HSQC

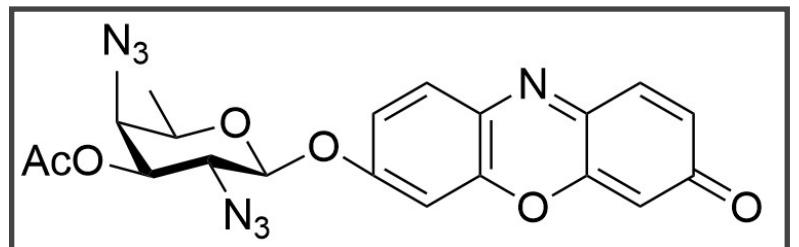

4

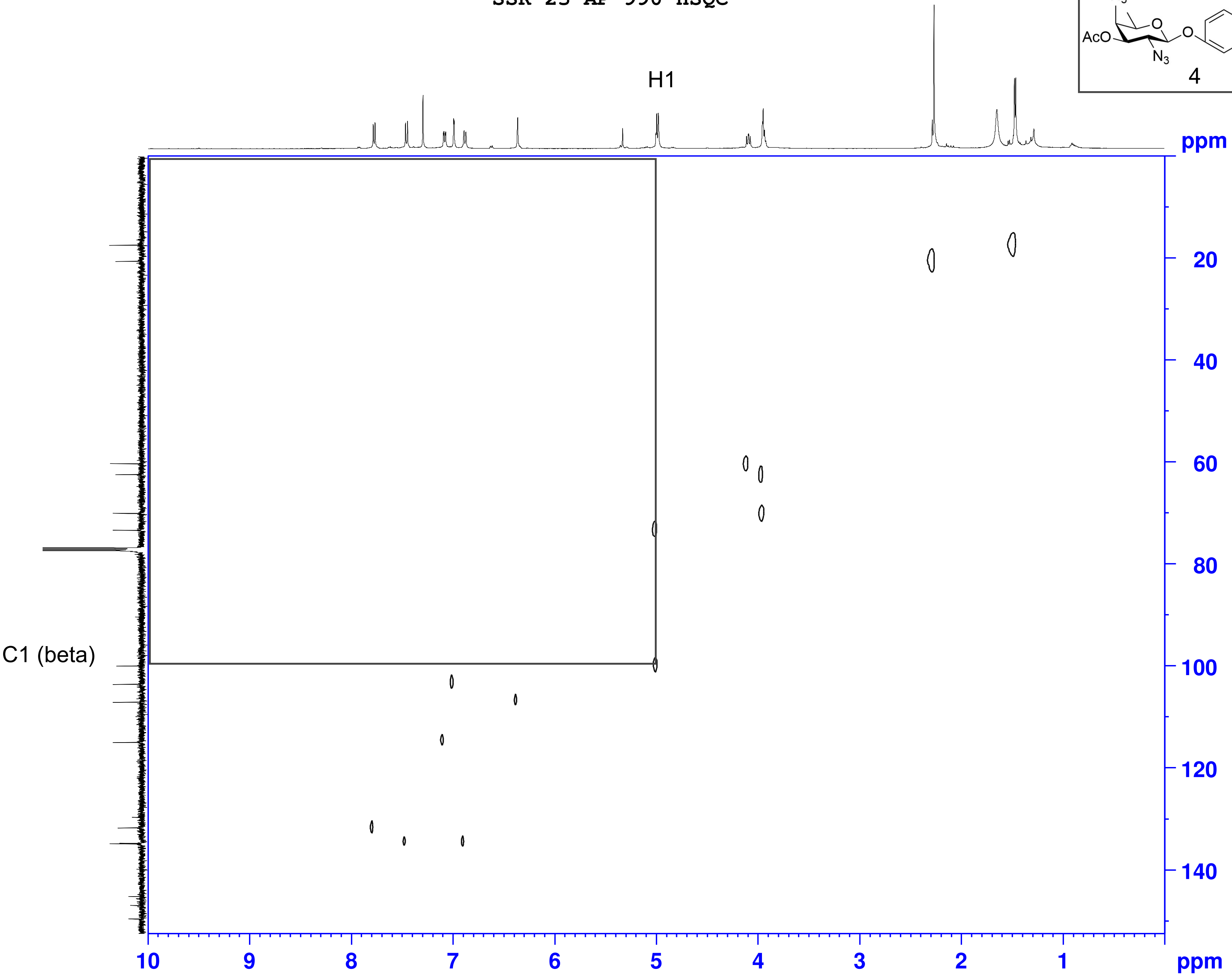


```
Current Data Parameters
NAME      SSK-23-AP-990-HSQC
EXPNO                    1
PROCNO                   1

F2 - Acquisition Parameters
Date_             20220207
Time                 12.41
INSTRUM              spect
PROBHD    5 mm PABBO BB/
PULPROG   hsqcedetgpsisp2.2
TD                    1024
SOLVENT              CDCl3
NS                       4
DS                       0
SWH               8012.820 Hz
FIDRES            7.825020 Hz
AQ               0.0638976 sec
RG                  197.27
DW                  62.400 usec
DE                    6.50 usec
TE                   297.1 K
CNST2          145.0000000
CNST17          -0.5000000
D0              0.00000300 sec
D1              1.00000000 sec
D4              0.00172414 sec
D11             0.03000000 sec
D16             0.00020000 sec
D21             0.00360000 sec
D24             0.00089000 sec
IN0             0.00002410 sec

======== CHANNEL f1 ========
SFO1           500.1323506 MHz
NUC1                    1H
P1                   13.35 usec
P2                   26.70 usec
P28                2000.00 usec
PLW1           16.00000000 W

======== CHANNEL f2 ========
SFO2           125.7665916 MHz
NUC2                   13C
CPDPRG[2 bi_p5m4sp_4sp.2
P3                    8.90 usec
P14                 500.00 usec
P24                2000.00 usec
P63                1500.00 usec
PLW0                     0 W
PLW2          103.00000000 W
PLW12           1.66499996 W
SPNAM[3] Crp60,0.5,20.1
SPOAL3               0.500
SPOFFS3                  0 Hz
SPW3           12.46500015 W
SPNAM[7]      Crp60comp.4
SPOAL7               0.500
SPOFFS7                  0 Hz
SPW7           12.46500015 W
SPNAM[14 Crp32,1.5,20.2
SPOAL14              0.500
SPOFFS14                 0 Hz
SPW14           5.31860018 W
SPNAM[31 Crp32,1.5,20.2
SPOAL31              0.500
SPOFFS31                 0 Hz
SPW31           1.32969999 W

====== GRADIENT CHANNEL =====
GPNAM[1]      SMSQ10.100
GPNAM[2]      SMSQ10.100
GPNAM[3]      SMSQ10.100
GPNAM[4]      SMSQ10.100
GPZ1                 80.00 %
GPZ2                 20.10 %
GPZ3                 11.00 %
GPZ4                 -5.00 %
P16                1000.00 usec
P19                 600.00 usec

F1 - Acquisition parameters
TD                      44
SFO1              125.7666 MHz
FIDRES          943.040344 Hz
SW                 164.963 ppm
FnMODE       Echo-Antiecho

F2 - Processing parameters
SI                    1024
SF             500.1300000 MHz
WDW                  QSINE
SSB                      2
LB                       0 Hz
GB                       0
PC                    1.40

F1 - Processing parameters
SI                    1024
MC2          echo-antiecho
SF             125.7577890 MHz
WDW                  QSINE
SSB                      2
LB                       0 Hz
GB                       0
```

SSK-23-AP-1009-1H

Current Data Parameters
NAME SSK-23-AP-1009-1H
EXPNO 10
PROCNO 1

F2 - Acquisition Parameters
Date_ 20220226
Time 23.37 h
INSTRUM Avance Neo 400
PROBHD Z163739_0226 (
PULPROG zg30
TD 51724
SOLVENT CDCl3
NS 18
DS 0
SWH 8620.689 Hz
FIDRES 0.333334 Hz
AQ 2.9999919 sec
RG 101
DW 58.000 usec
DE 13.14 usec
TE 298.6 K
D1 1.00000000 sec
TD0 1
SFO1 400.1324708 MHz
NUC1 1H
P0 2.67 usec
P1 8.00 usec
PLW1 25.07999992 W

F2 - Processing parameters
SI 65536
SF 400.1300000 MHz
WDW EM
SSB 0
LB 0.30 Hz
GB 0
PC 1.00

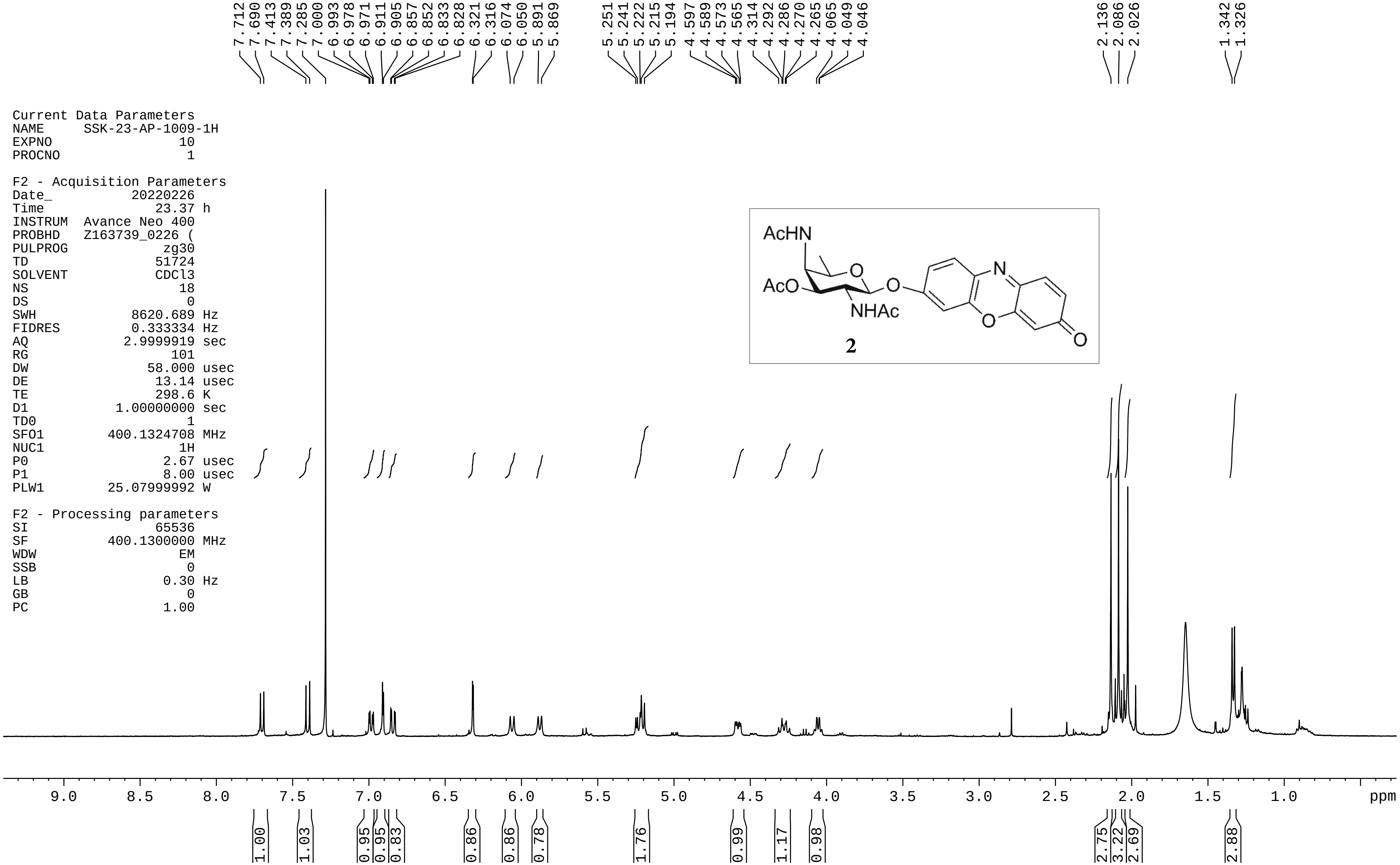

SSK-23-AP-1009-13C

186.31
171.04
170.77
170.61
160.31
149.47
146.68
145.11
134.78
134.58
131.69
129.44
114.67
107.04
103.24
99.48
70.84
70.50
51.01
50.19
23.43
23.24
20.84
16.69

```
Current Data Parameters
NAME     SSK-23-AP-1009-13C
EXPNO               16
PROCNO               1

F2 - Acquisition Parameters
Date_          20220227
Time               7.58 h
INSTRUM  Avance Neo 400
PROBHD   Z163739_0226 (
PULPROG          zgpg30
TD                65536
SOLVENT           CDCl3
NS                 8550
DS                    0
SWH           27777.777 Hz
FIDRES         0.847710 Hz
AQ            1.1796480 sec
RG                  101
DW               18.000 usec
DE                 6.50 usec
TE                302.8 K
D1           1.00000000 sec
D11          0.03000000 sec
TD0                   1
SFO1        100.6242384 MHz
NUC1                13C
P0                 2.67 usec
P1                 8.00 usec
PLW1        99.33999634 W
SFO2        400.1316005 MHz
NUC2                 1H
CPDPRG[2         waltz65
PCPD2             90.00 usec
PLW2        25.07999992 W
PLW12        0.19815999 W
PLW13        0.09967500 W

F2 - Processing parameters
SI                32768
SF          100.6127685 MHz
WDW                  EM
SSB                   0
LB                 1.00 Hz
GB                    0
PC                 1.40
```

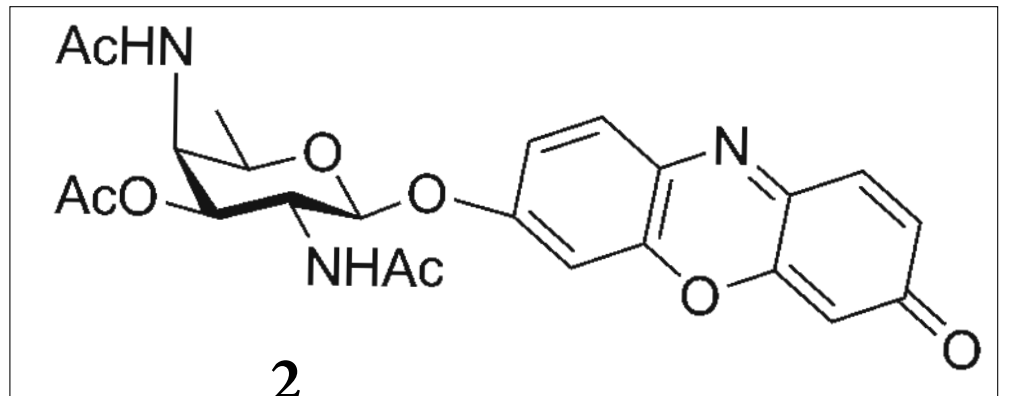

2

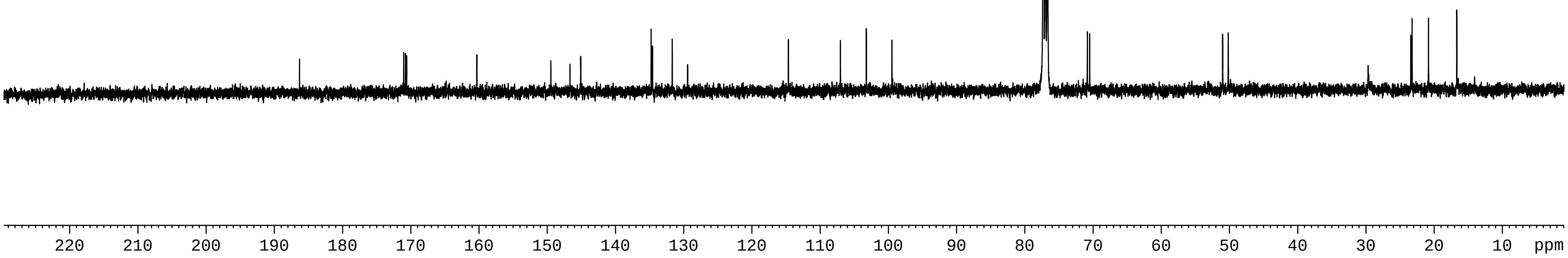

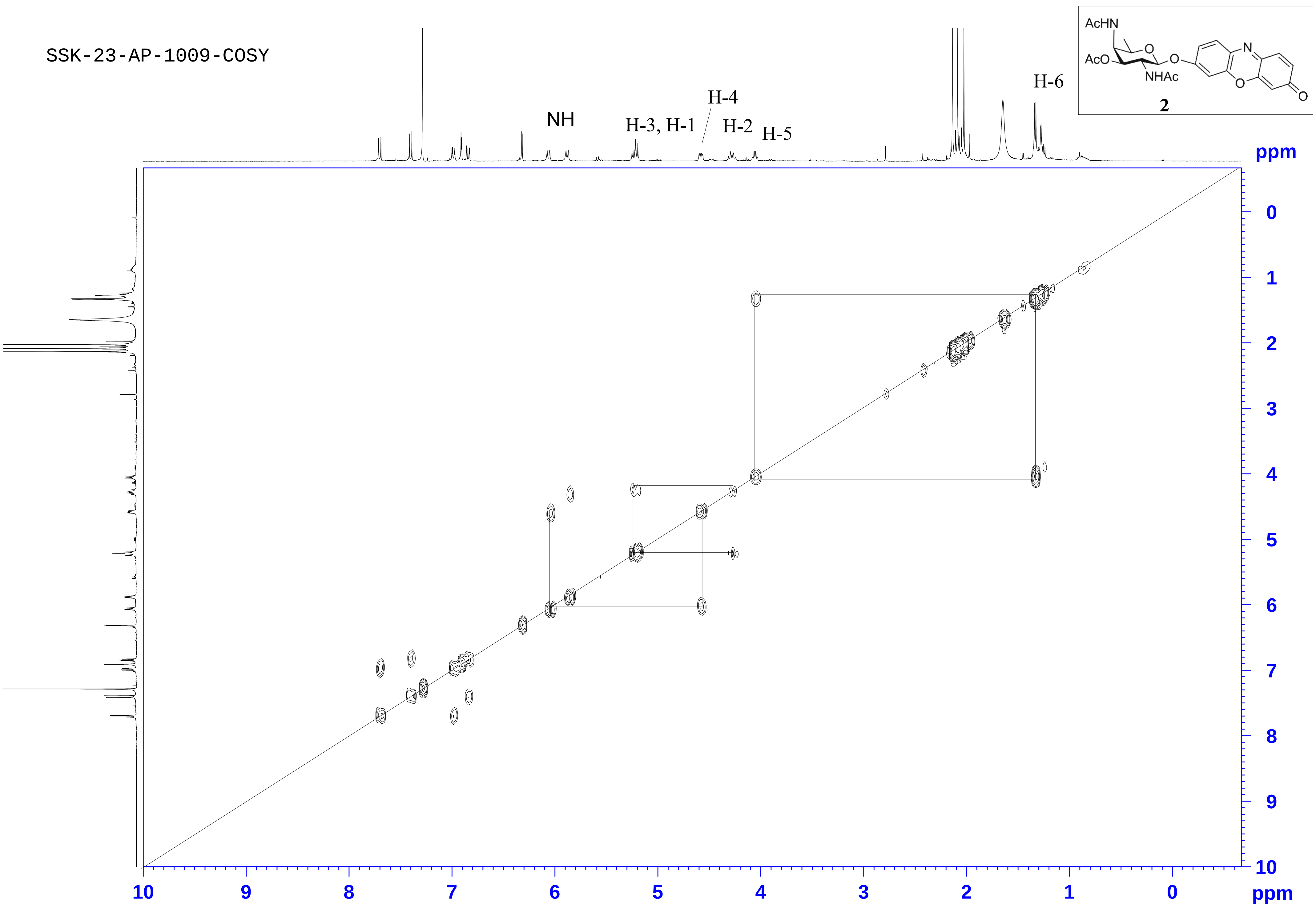
SSK-23-AP-1009-COSY
NH
H-3, H-1
H-4
H-2
H-5
H-6
AcHN
AcO
O
NHAc
N
O
O
2
ppm
0
1
2
3
4
5
6
7
8
9
10
ppm

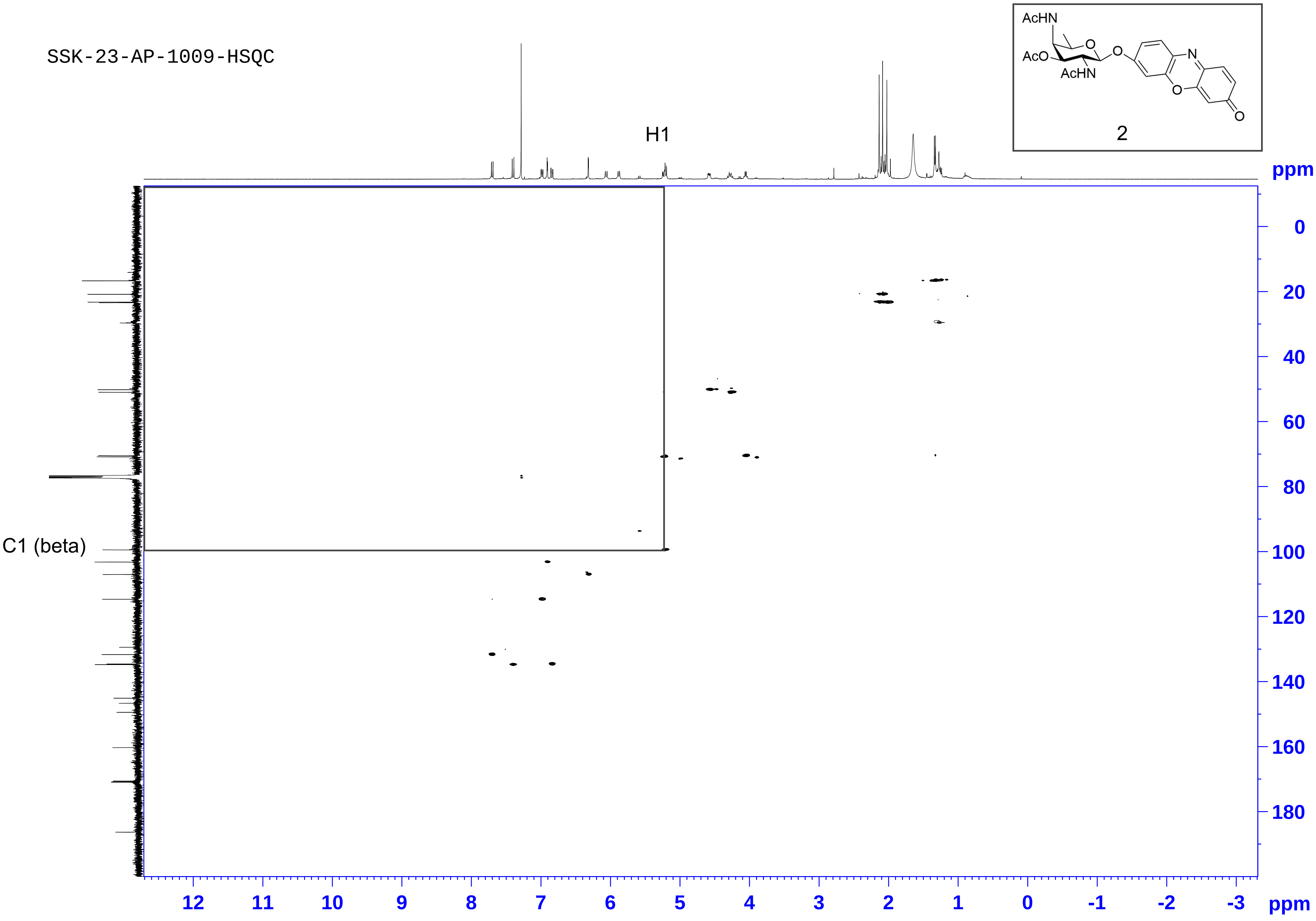
SSK-23-AP-1009-HSQC
H1
C1 (beta)
AcHN
AcO
AcHN
O
O
N
O
O
2
ppm
0
20
40
60
80
100
120
140
160
180
12
11
10
9
8
7
6
5
4
3
2
1
0
-1
-2
-3
ppm

SSK-23-AP-1003-1H

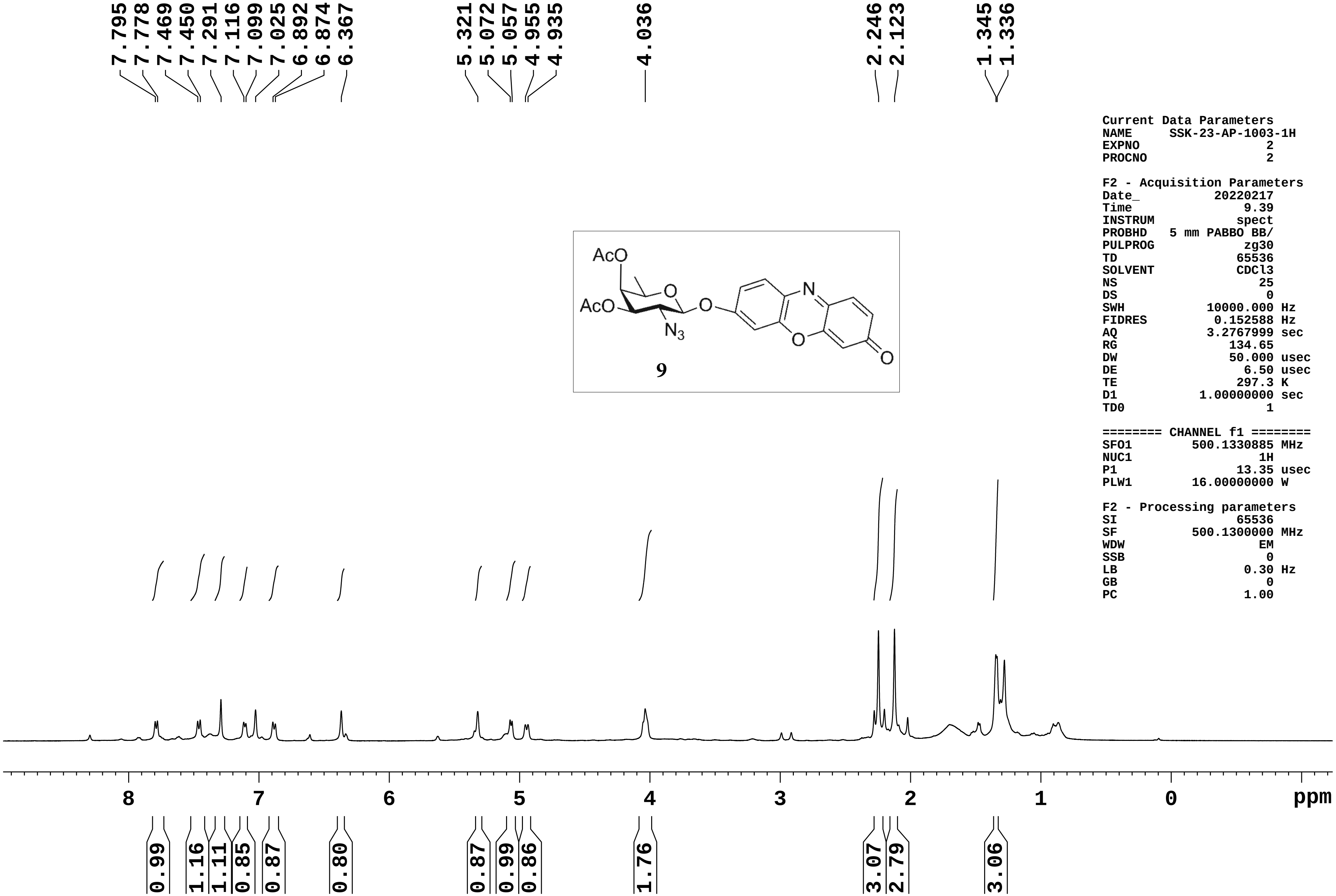

SSK-23-AP-1003-13C

```
Current Data Parameters
NAME      SSK-23-AP-1003-13C
EXPNO                    2
PROCNO                   2

F2 - Acquisition Parameters
Date_           20220217
Time               10.18
INSTRUM            spect
PROBHD   5 mm PABBO BB/
PULPROG          zgpg30
TD                 65536
SOLVENT            CDCl3
NS                  1000
DS                     0
SWH           29761.904 Hz
FIDRES         0.454131 Hz
AQ            1.1010048 sec
RG                197.27
DW                16.800 usec
DE                  6.50 usec
TE                 297.0 K
D1           1.00000000 sec
D11          0.03000000 sec
TD0                    1

======== CHANNEL f1 ========
SFO1        125.7703637 MHz
NUC1                 13C
P1                  8.90 usec
PLW1       103.00000000 W

======== CHANNEL f2 ========
SFO2        500.1320005 MHz
NUC2                  1H
CPDPRG[2         waltz16
PCPD2              80.00 usec
PLW2         16.00000000 W
PLW12         0.44556001 W
PLW13         0.22411001 W

F2 - Processing parameters
SI                 32768
SF          125.7577890 MHz
WDW                   EM
SSB                    0
LB                  1.00 Hz
GB                     0
PC                  1.40
```

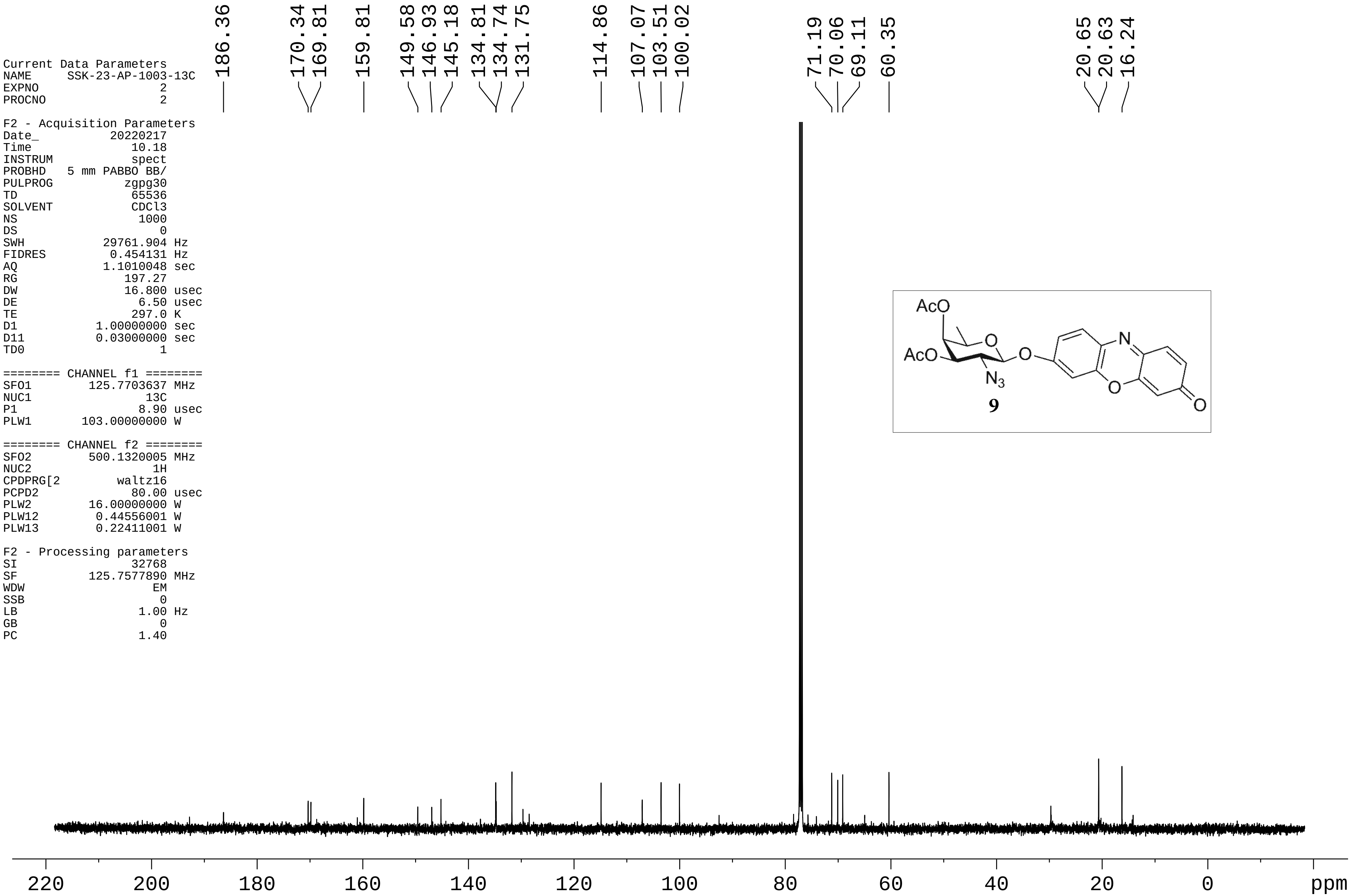

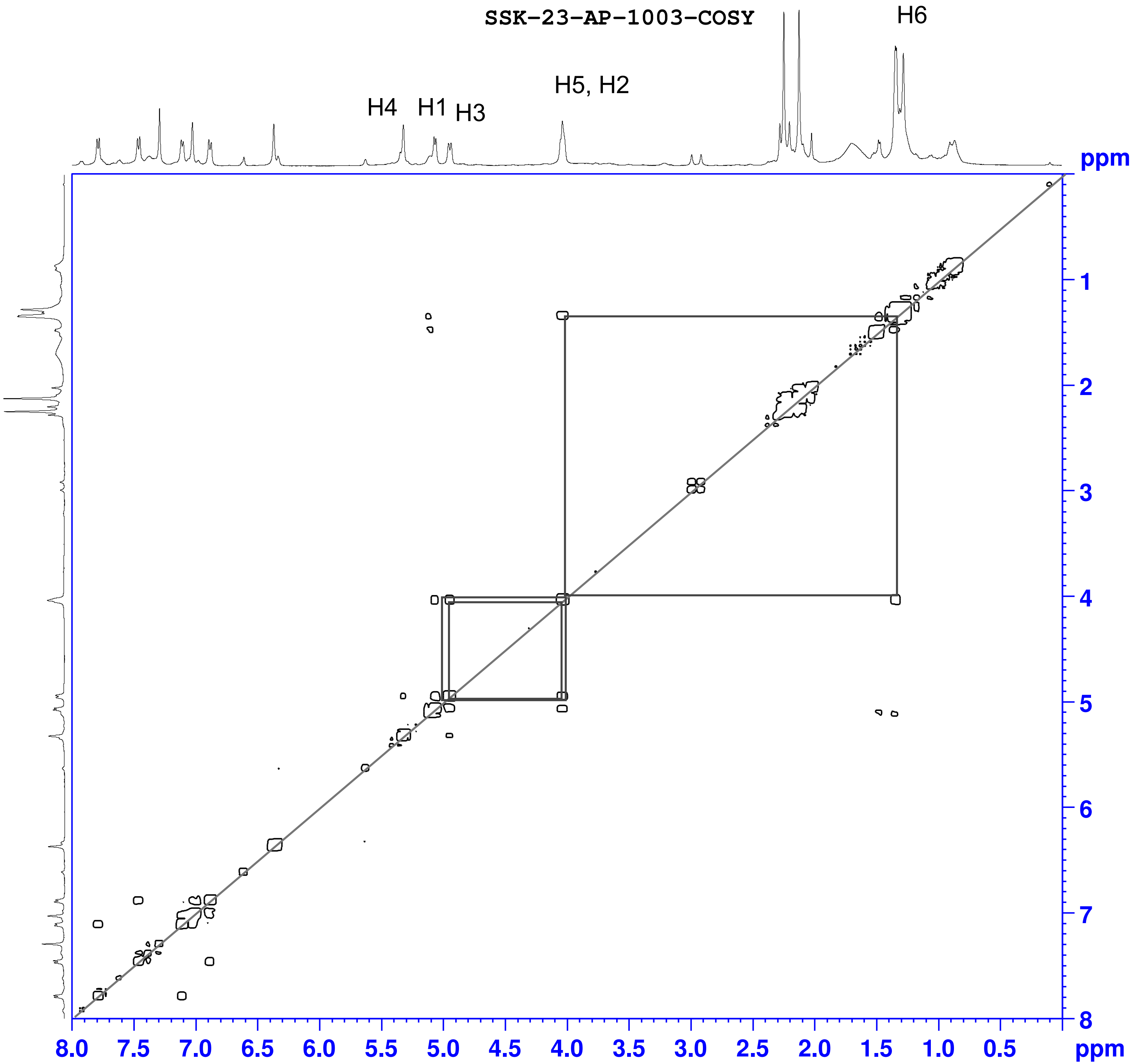


```
Current Data Parameters
NAME       SSK-23-AP-1003-COSY
EXPNO                    2
PROCNO                   2

F2 - Acquisition Parameters
Date_             20220217
Time                 10.47
INSTRUM              spect
PROBHD    5 mm PABBO BB/
PULPROG         cosygpppqf
TD                    2048
SOLVENT              CDCl3
NS                       4
DS                       0
SWH               6684.492 Hz
FIDRES            3.263912 Hz
AQ               0.1531904 sec
RG                   61.42
DW                  74.800 usec
DE                    6.50 usec
TE                   297.7 K
D0              0.00000300 sec
D1              1.00000000 sec
D11             0.03000000 sec
D12             0.00002000 sec
D13             0.00000400 sec
D16             0.00020000 sec
IN0             0.00014960 sec

======== CHANNEL f1 ========
SFO1           500.1330069 MHz
NUC1                    1H
P0                   13.35 usec
P1                   13.35 usec
P17                5000.00 usec
PLW1           16.00000000 W
PLW10           3.16840005 W

====== GRADIENT CHANNEL =====
GPNAM[1]        SMSQ10.100
GPZ1                 10.00 %
P16                1000.00 usec

F1 - Acquisition parameters
TD                      73
SFO1               500.133 MHz
FIDRES          183.136765 Hz
SW                  13.365 ppm
FnMODE                  QF

F2 - Processing parameters
SI                    1024
SF             500.1300000 MHz
WDW                  QSINE
SSB                      0
LB                       0 Hz
GB                       0
PC                    1.40

F1 - Processing parameters
SI                    1024
MC2                     QF
SF             500.1300000 MHz
WDW                  QSINE
SSB                      0
LB                       0 Hz
GB                       0
```

SSK-23-AP-1003-HSQC n

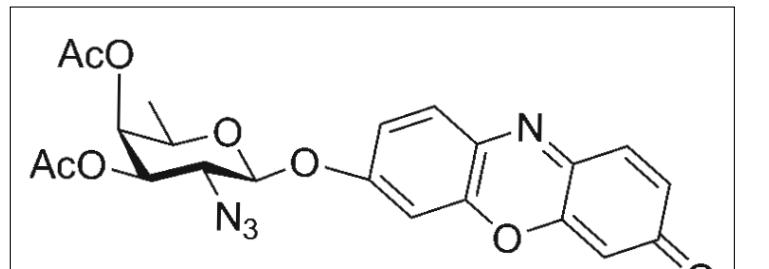

9

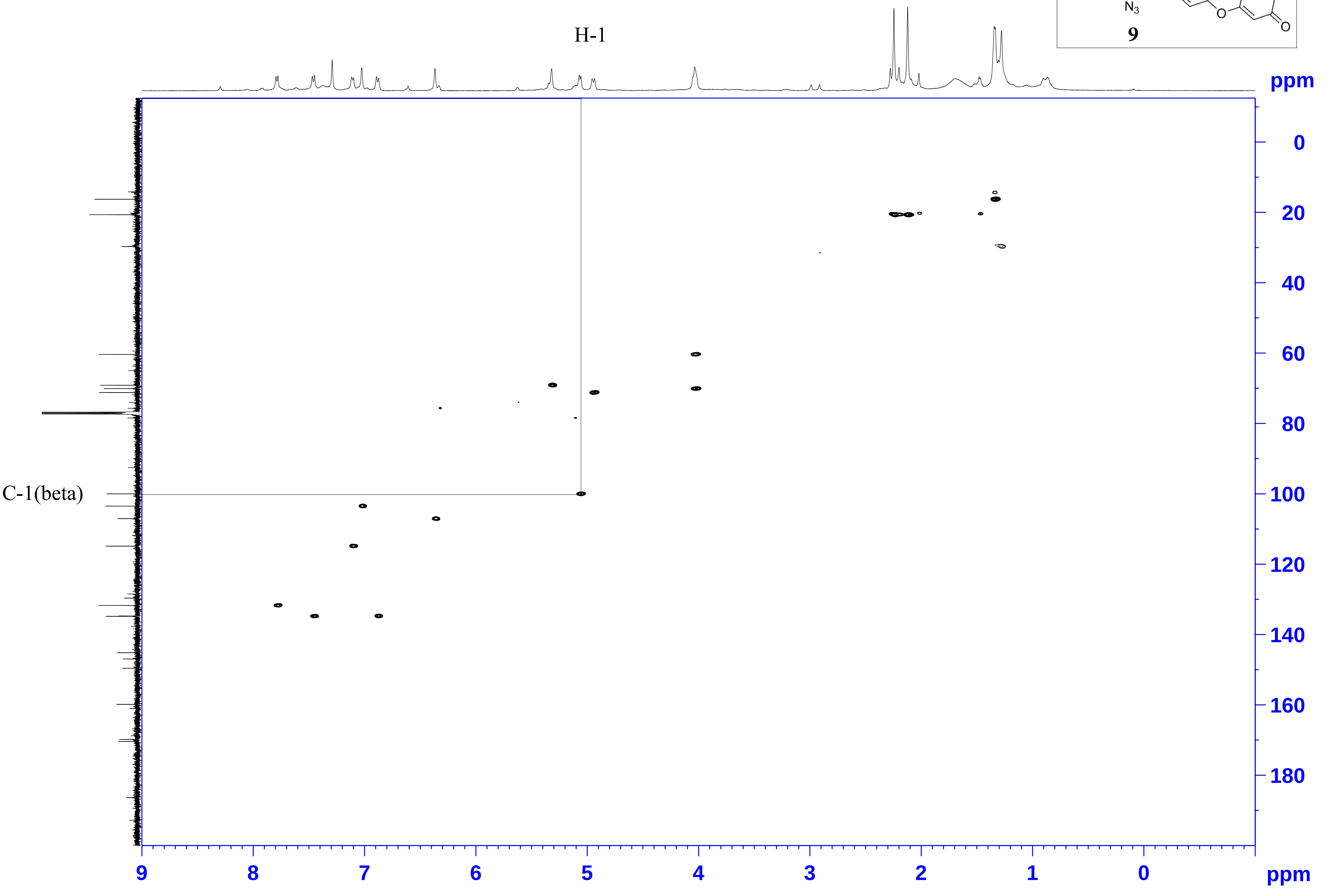

SSK-23-AP-1004-2-1H

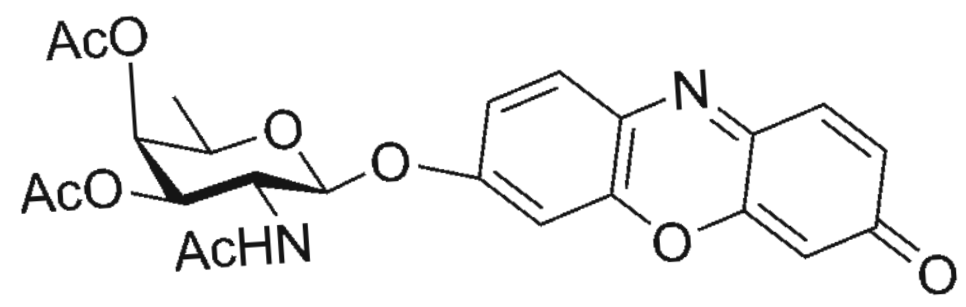

3

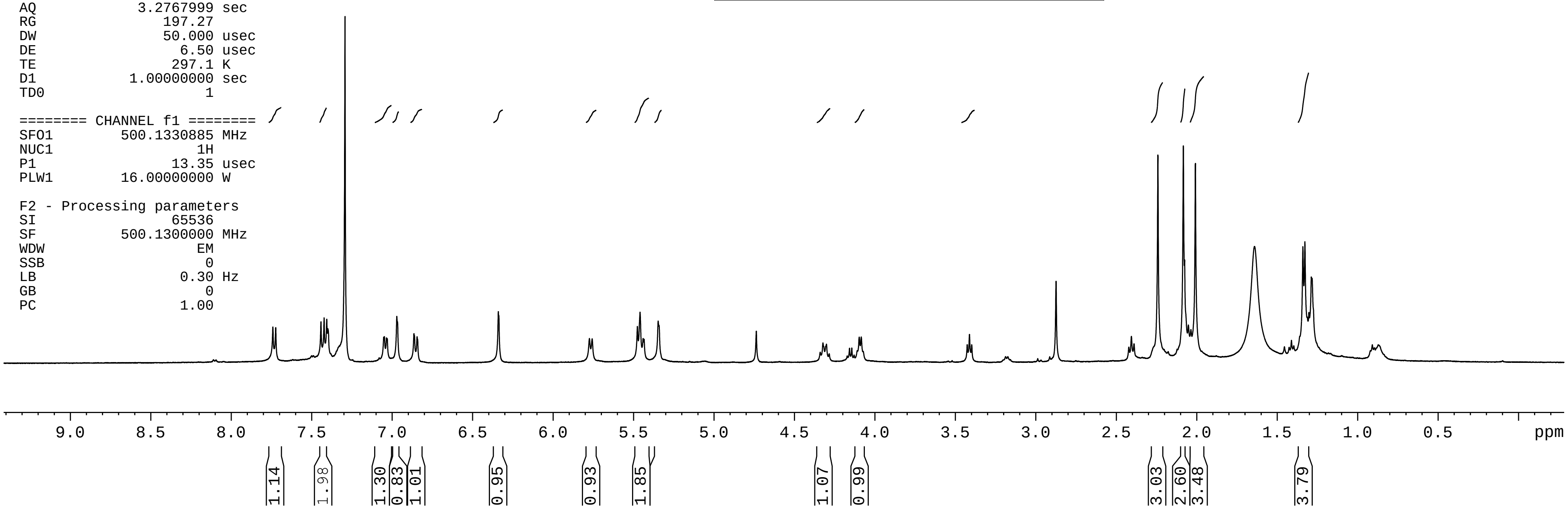

SSK-23-AP-1004-2-13C

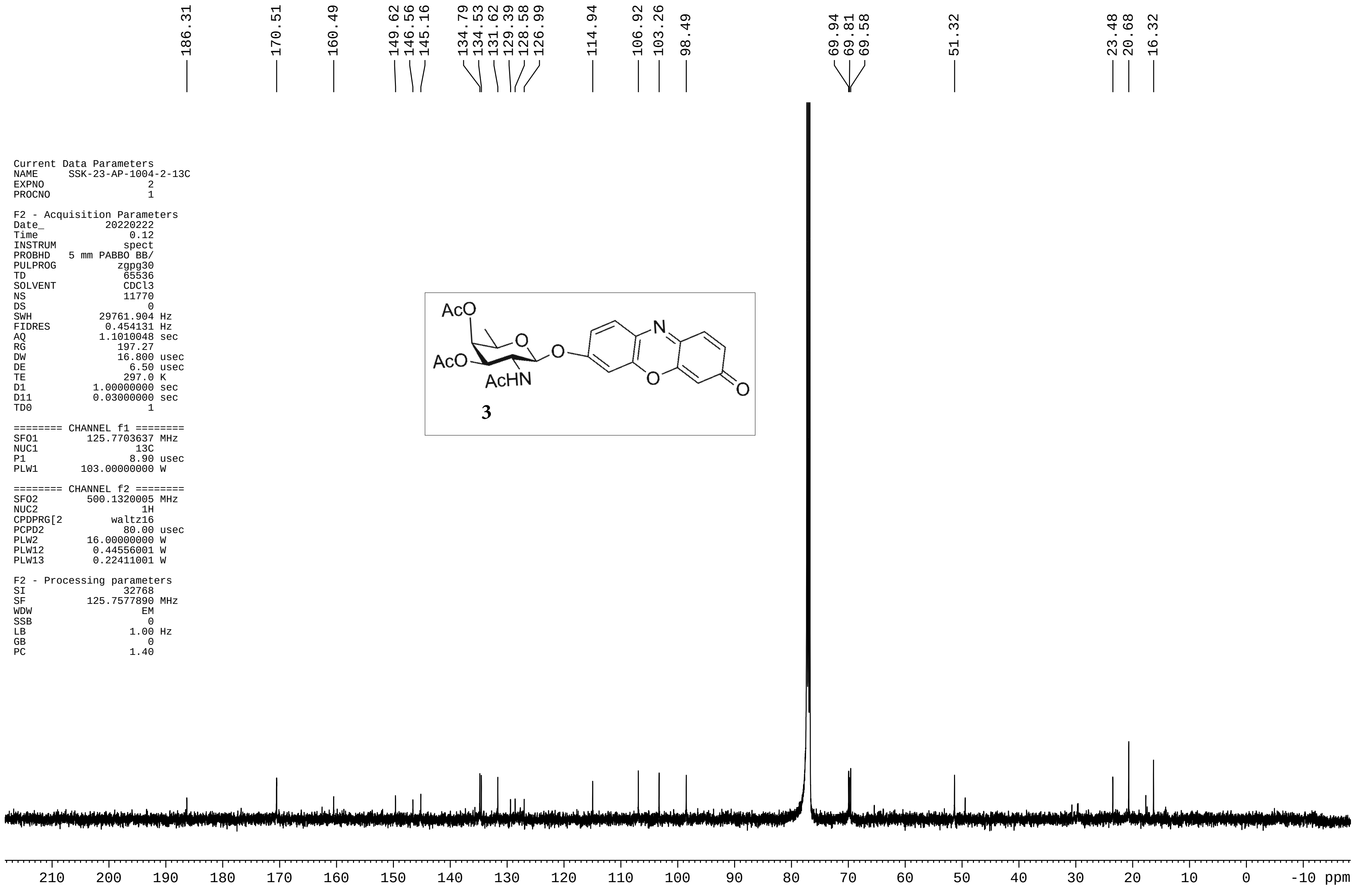


```
Current Data Parameters
NAME     SSK-23-AP-1004-2-13C
EXPNO                   2
PROCNO                  1

F2 - Acquisition Parameters
Date_          20220222
Time               0.12
INSTRUM           spect
PROBHD   5 mm PABBO BB/
PULPROG          zgpg30
TD                65536
SOLVENT           CDCl3
NS                11770
DS                    0
SWH           29761.904 Hz
FIDRES         0.454131 Hz
AQ            1.1010048 sec
RG               197.27
DW               16.800 usec
DE                 6.50 usec
TE                297.0 K
D1           1.00000000 sec
D11          0.03000000 sec
TD0                   1

======== CHANNEL f1 ========
SFO1        125.7703637 MHz
NUC1                13C
P1                 8.90 usec
PLW1       103.00000000 W

======== CHANNEL f2 ========
SFO2        500.1320005 MHz
NUC2                 1H
CPDPRG[2        waltz16
PCPD2             80.00 usec
PLW2        16.00000000 W
PLW12        0.44556001 W
PLW13        0.22411001 W

F2 - Processing parameters
SI                32768
SF          125.7577890 MHz
WDW                  EM
SSB                   0
LB                 1.00 Hz
GB                    0
PC                 1.40
```


3

SSK-23-AP-1004-2-COSY

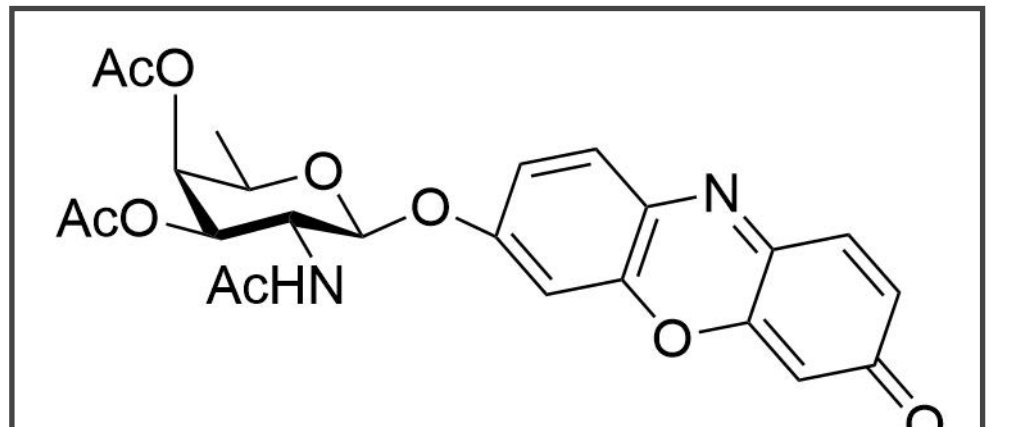

2

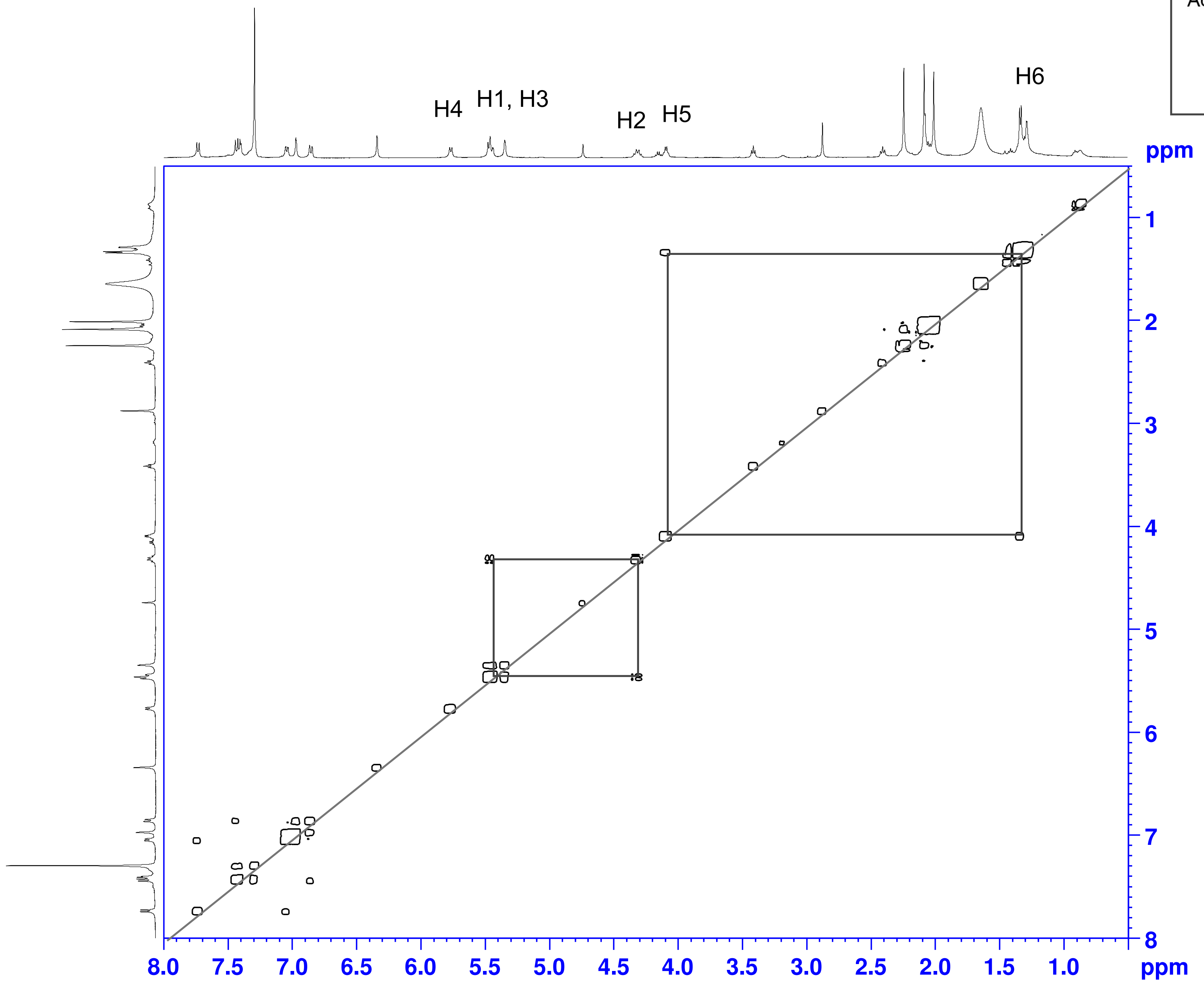


```
Current Data Parameters
NAME       SSK-23-AP-1004-2-COSY
EXPNO                   2
PROCNO                  1

F2 - Acquisition Parameters
Date_            20220221
Time                23.57
INSTRUM             spect
PROBHD   5 mm PABBO BB/
PULPROG         cosygpppqf
TD                   2048
SOLVENT             CDCl3
NS                      8
DS                      0
SWH             6684.492 Hz
FIDRES          3.263912 Hz
AQ             0.1531904 sec
RG                  61.42
DW                 74.800 usec
DE                   6.50 usec
TE                  297.0 K
D0            0.00000300 sec
D1            1.00000000 sec
D11           0.03000000 sec
D12           0.00002000 sec
D13           0.00000400 sec
D16           0.00020000 sec
IN0           0.00014960 sec

======== CHANNEL f1 ========
SFO1         500.1330069 MHz
NUC1                  1H
P0                 13.35 usec
P1                 13.35 usec
P17              5000.00 usec
PLW1         16.00000000 W
PLW10         3.16840005 W

====== GRADIENT CHANNEL =====
GPNAM[1]       SMSQ10.100
GPZ1               10.00 %
P16              1000.00 usec

F1 - Acquisition parameters
TD                     59
SFO1             500.133 MHz
FIDRES        226.592941 Hz
SW                13.365 ppm
FnMODE                 QF

F2 - Processing parameters
SI                   1024
SF           500.1300000 MHz
WDW                 QSINE
SSB                     0
LB                      0 Hz
GB                      0
PC                   1.40

F1 - Processing parameters
SI                   1024
MC2                    QF
SF           500.1300000 MHz
```

SSK-23-AP-1004-2-HSQC

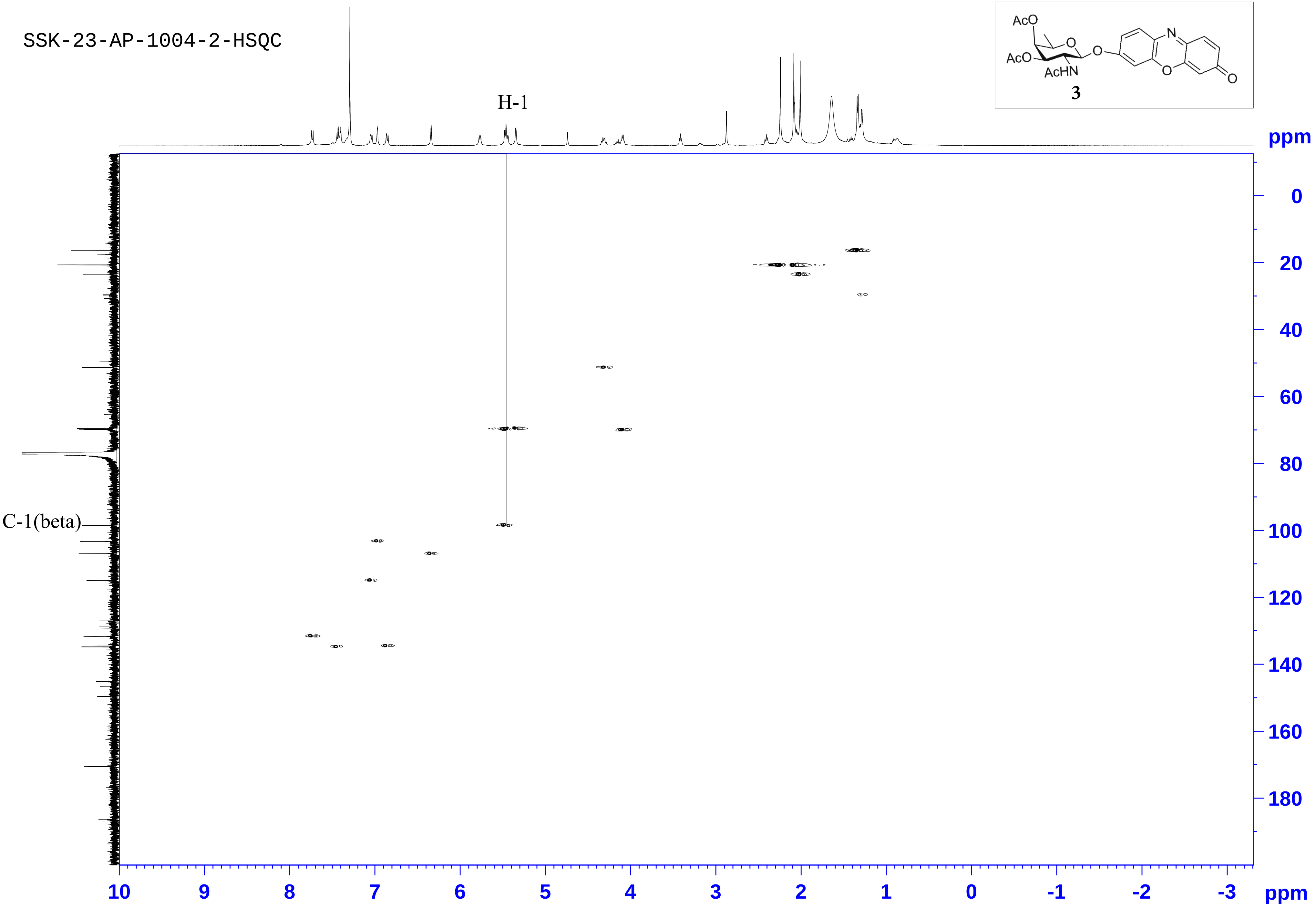

SSK-32-SKM-GLC-OAc-RES-1H

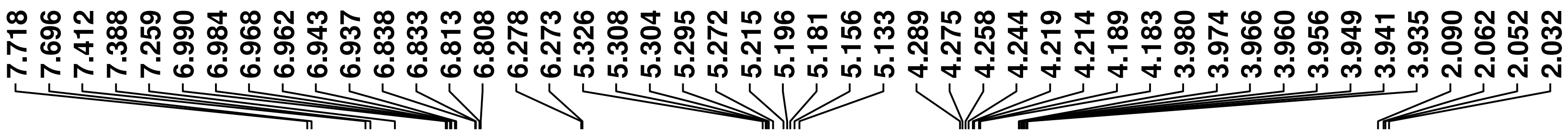

Current Data Parameters
NAME SSK-32-SKM-GLC-OAc-RES-1H
EXPNO 82
PROCNO 1

F2 - Acquisition Parameters
Date_ 20260523
Time 22.43 h
INSTRUM spect
PROBHD Z104450_0346 (
PULPROG zg30
TD 54274
SOLVENT CDCl3
NS 19
DS 0
SWH 8223.685 Hz
FIDRES 0.303043 Hz
AQ 3.2998593 sec
RG 161
DW 60.800 usec
DE 6.50 usec
TE 158.7 K
D1 1.00000000 sec
TD0 1
SFO1 400.1324710 MHz
NUC1 1H
P0 5.00 usec
P1 15.00 usec
PLW1 9.69999981 W

F2 - Processing parameters
SI 32768
SF 400.1300095 MHz
WDW EM
SSB 0
LB 0.30 Hz
GB 0
PC 1.00

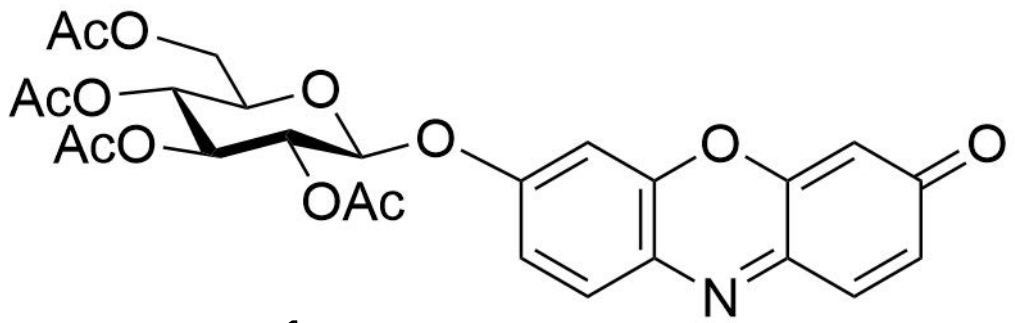

1a

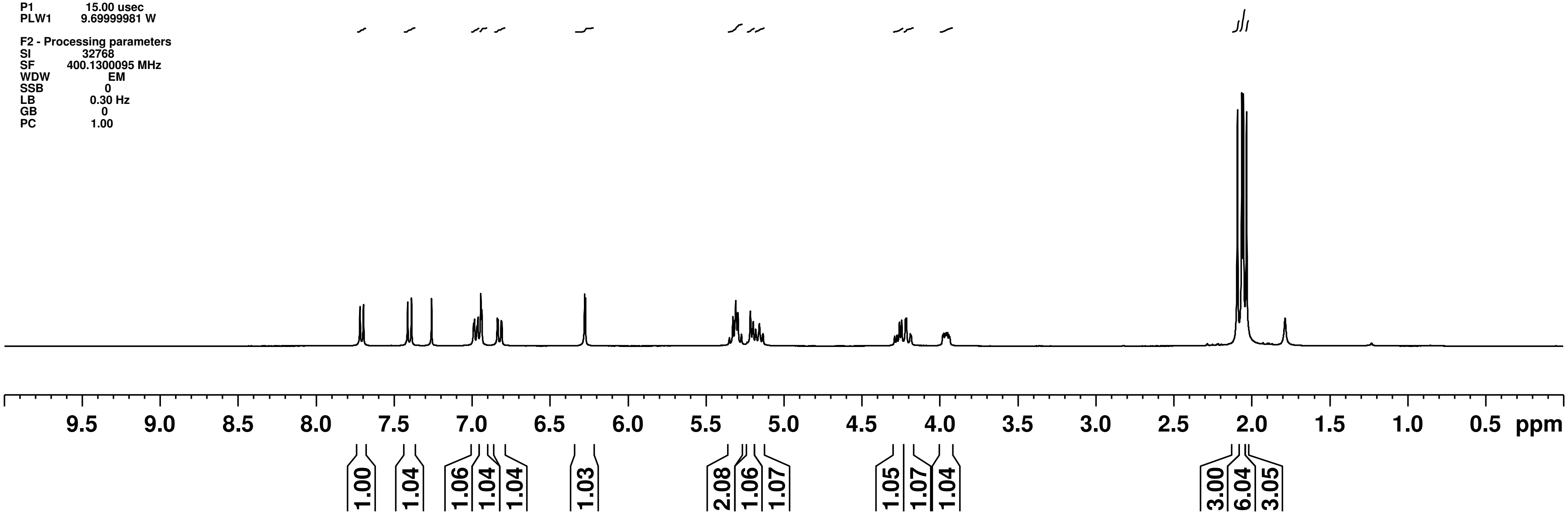

SSK-32-SKM-GLC-OAC-RES-13C

186.25, 170.43, 170.16, 169.40, 169.21, 159.77, 149.49, 146.90, 145.08, 134.79, 134.70, 131.61, 129.57, 114.99, 106.95, 103.42, 98.31, 72.46, 70.91, 68.07, 61.91, 20.65, 20.63, 20.59

```
Current Data Parameters
NAME     SSK-32-SKM-GLC-OAC-RES-13C
EXPNO                    11
PROCNO                    1

F2 - Acquisition Parameters
Date_          20260524
Time              10.30 h
INSTRUM           spect
PROBHD   Z104450_0346 (
PULPROG         zgpg30
TD                65536
SOLVENT           CDCl3
NS                   91
DS                    0
SWH           26041.666 Hz
FIDRES         0.794729 Hz
AQ            1.2582912 sec
RG                 1030
DW               19.200 usec
DE                 6.50 usec
TE                158.5 K
D1           1.00000000 sec
D11          0.03000000 sec
TD0                   1
SFO1        100.6238364 MHz
NUC1                13C
P0                 3.33 usec
P1                10.00 usec
PLW1        47.00000000 W
SFO2        400.1316005 MHz
NUC2                 1H
CPDPRG[2        waltz16
PCPD2             90.00 usec
PLW2         9.69999981 W
PLW12        0.26944000 W
PLW13        0.13552999 W

F2 - Processing parameters
SI                32768
SF          100.6127690 MHz
WDW                  EM
SSB                   0
LB                 1.00 Hz
GB                    0
PC                 1.40
```

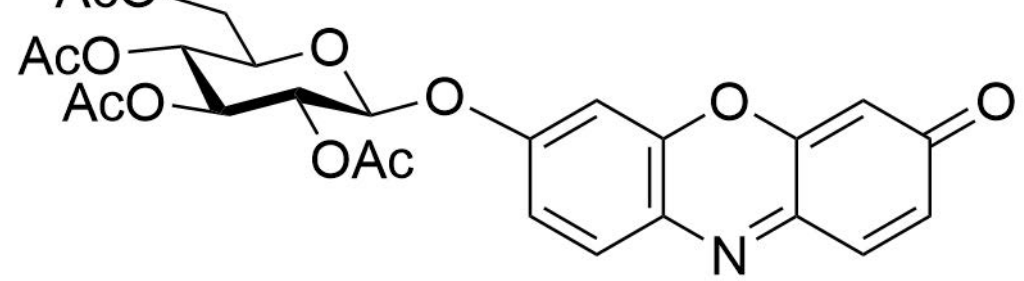

1a

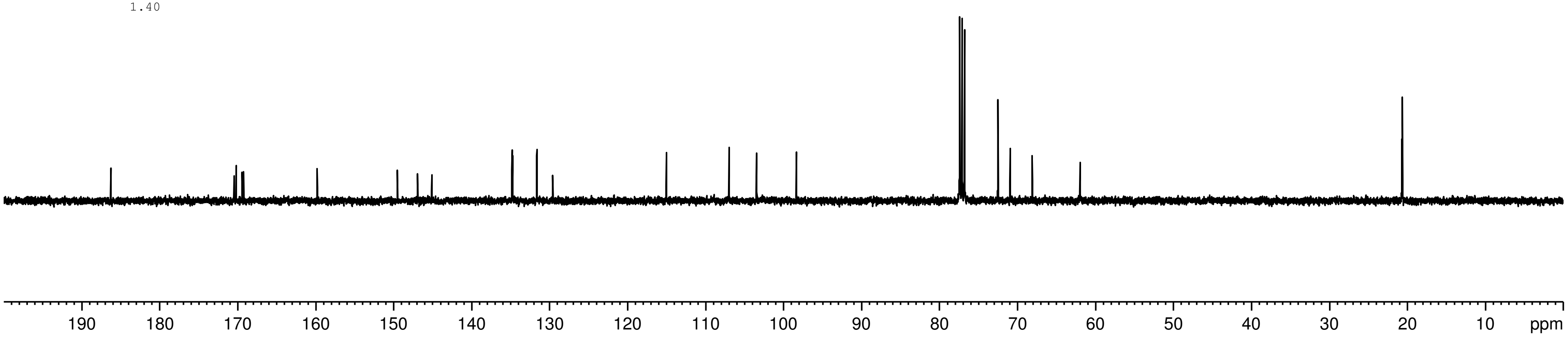

SSK-32-SKM-GLC-OAC-RES-DEPT

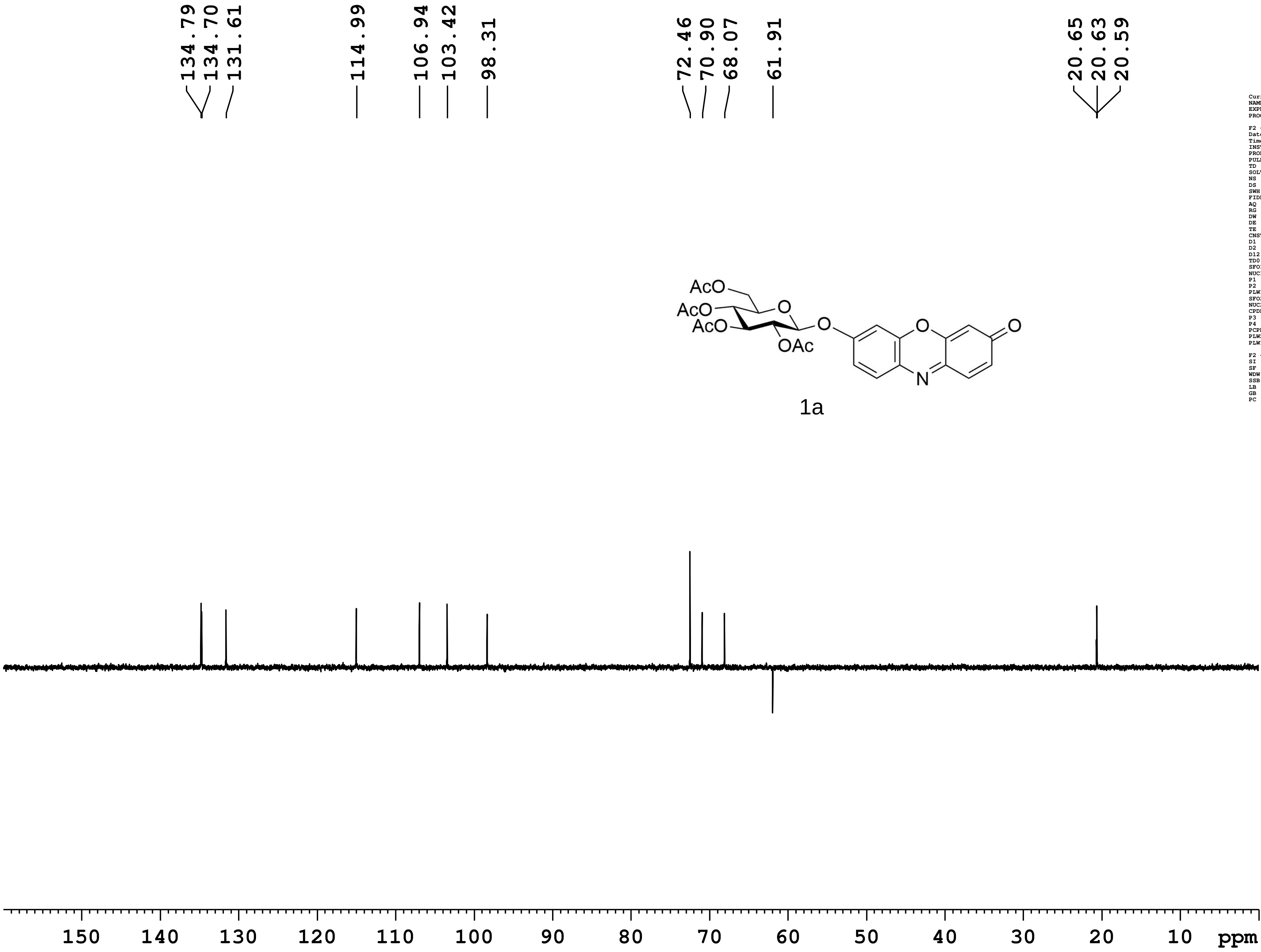

1a

```
Current Data Parameters
NAME      SSK-32-SKM-GLC-OAC-RES-DEPT
EXPNO                    12
PROCNO                    1

F2 - Acquisition Parameters
Date_              20260524
Time                  10.34 h
INSTRUM               spect
PROBHD     Z104450_0346 (
PULPROG             dept135
TD                    65536
SOLVENT               CDCl3
NS                       60
DS                        1
SWH               27777.777 Hz
FIDRES             0.847710 Hz
AQ                1.1796480 sec
RG                      203
DW                   18.000 usec
DE                     6.50 usec
TE                    158.4 K
CNST2           145.0000000
D1               2.00000000 sec
D2               0.00344828 sec
D12              0.00002000 sec
TD0                       1
SFO1            100.6242389 MHz
NUC1                    13C
P1                    10.00 usec
P2                    20.00 usec
PLW1            47.00000000 W
SFO2            400.1316005 MHz
NUC2                     1H
CPDPRG[2            waltz16
P3                    15.00 usec
P4                    30.00 usec
PCPD2                 90.00 usec
PLW2             9.69999981 W
PLW12            0.26944000 W

F2 - Processing parameters
SI                    32768
SF              100.6127690 MHz
WDW                      EM
SSB                       0
LB                     1.00 Hz
GB                        0
PC                     1.40
```

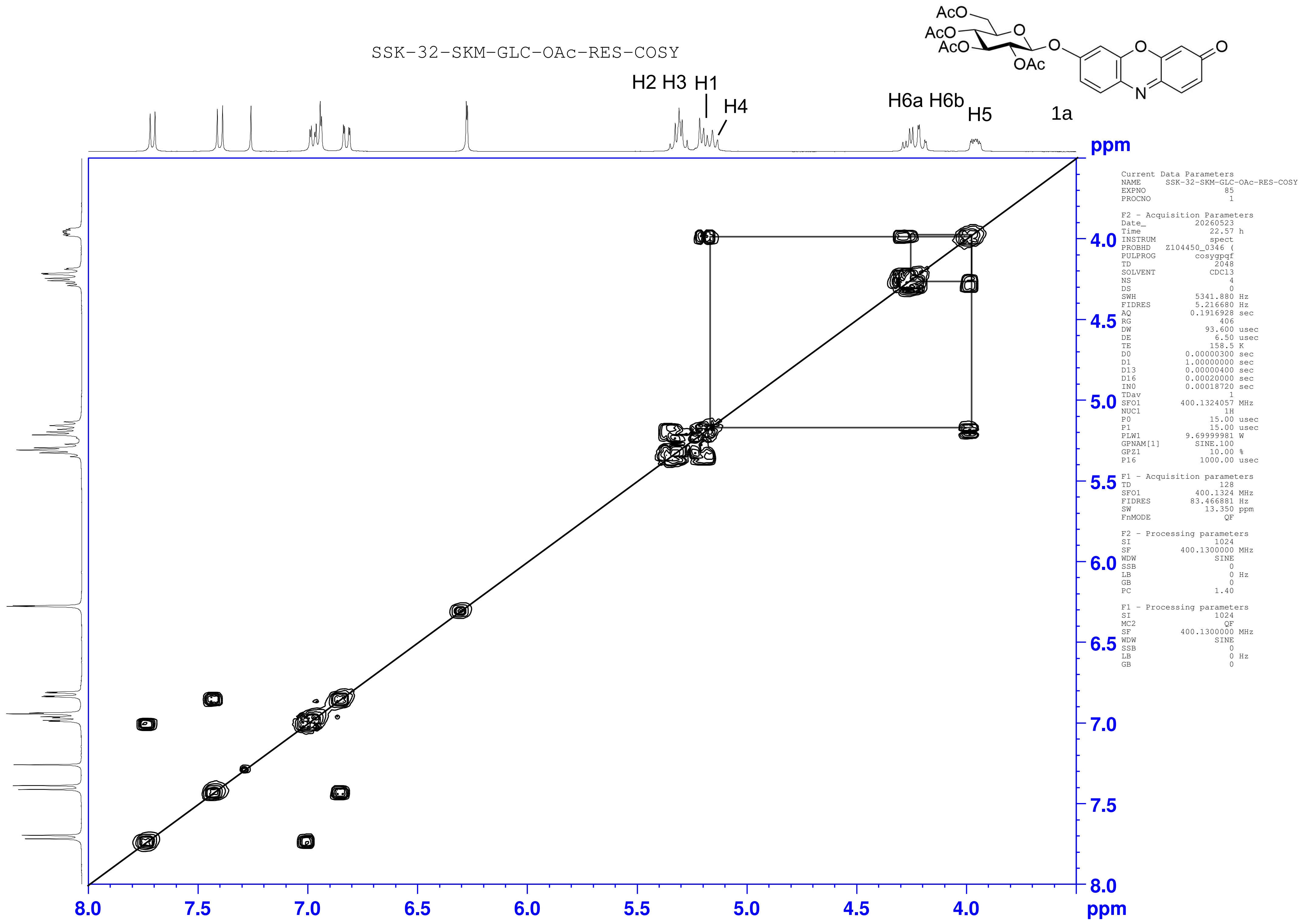
SSK-32-SKM-GLC-OAc-RES-COSY
AcO
AcO
AcO
O
O
OAc
O
O
N
O
1a
H2 H3
H1
H4
H6a H6b
H5
ppm
8.0
7.5
7.0
6.5
6.0
5.5
5.0
4.5
4.0
ppm
Current Data Parameters
NAME SSK-32-SKM-GLC-OAc-RES-COSY
EXPNO 85
PROCNO 1
F2 - Acquisition Parameters
Date_ 20260523
Time 22.57 h
INSTRUM spect
PROBHD Z104450_0346 (
PULPROG cosygpqf
TD 2048
SOLVENT CDCl3
NS 4
DS 0
SWH 5341.880 Hz
FIDRES 5.216680 Hz
AQ 0.1916928 sec
RG 406
DW 93.600 usec
DE 6.50 usec
TE 158.5 K
D0 0.00000300 sec
D1 1.00000000 sec
D13 0.00000400 sec
D16 0.00020000 sec
IN0 0.00018720 sec
TDav 1
SFO1 400.1324057 MHz
NUC1 1H
P0 15.00 usec
P1 15.00 usec
PLW1 9.69999981 W
GPNAM[1] SINE.100
GPZ1 10.00 %
P16 1000.00 usec
F1 - Acquisition parameters
TD 128
SFO1 400.1324 MHz
FIDRES 83.466881 Hz
SW 13.350 ppm
FnMODE QF
F2 - Processing parameters
SI 1024
SF 400.1300000 MHz
WDW SINE
SSB 0
LB 0 Hz
GB 0
PC 1.40
F1 - Processing parameters
SI 1024
MC2 QF
SF 400.1300000 MHz
WDW SINE
SSB 0
LB 0 Hz
GB 0

SSK-32-SKM-GLC-OAC-RES-HSQC

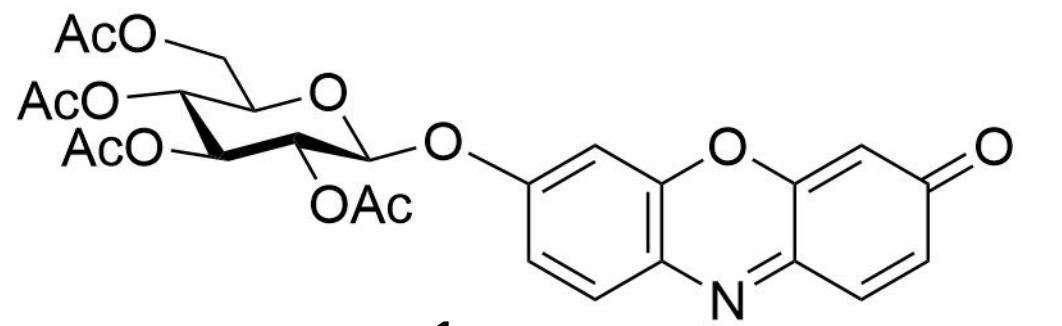

1a

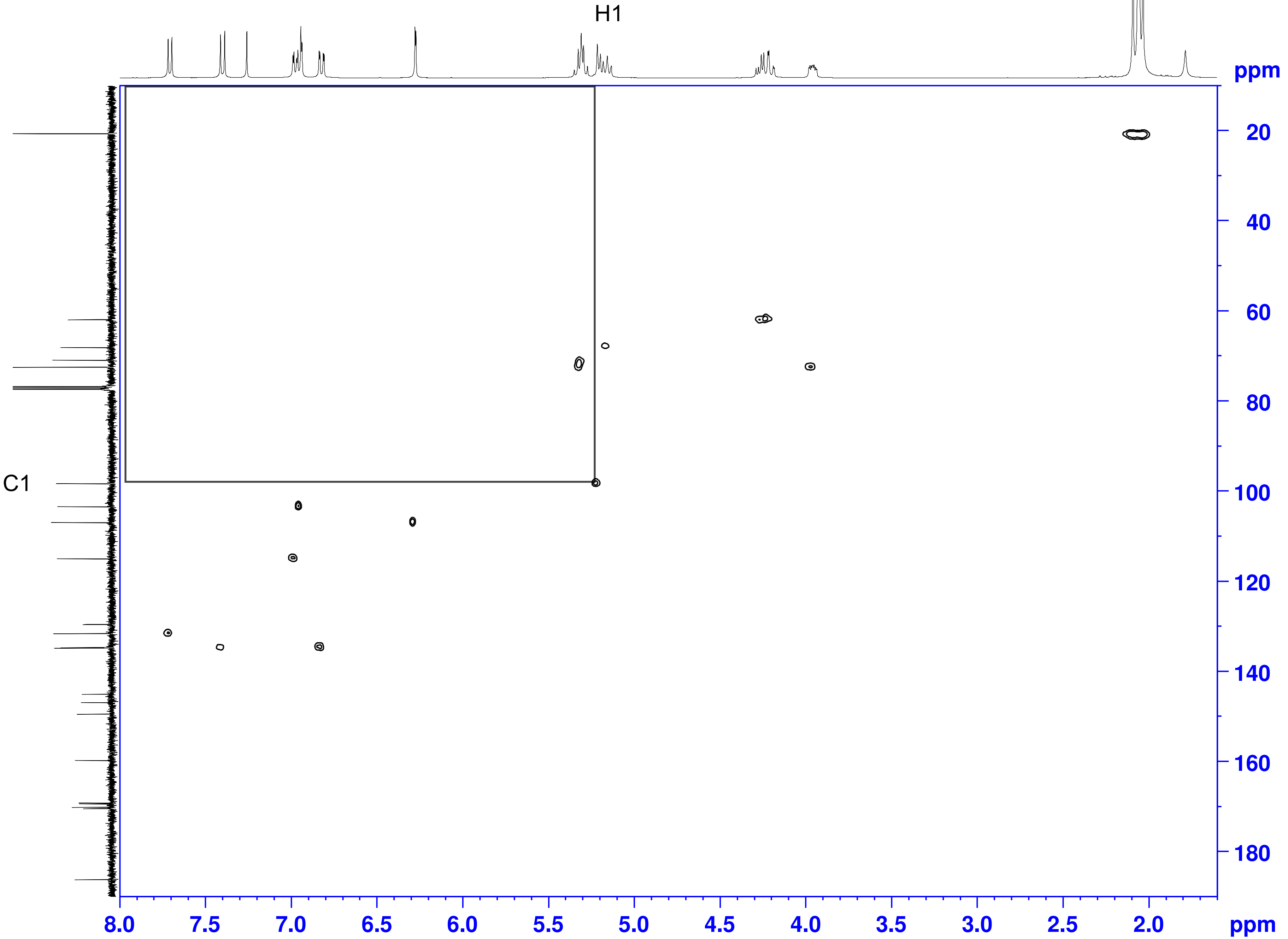

Current Data Parameters
NAME SSK-32-SKM-GLC-OAC-RES-HSQC
EXPNO 13
PROCNO 1

F2 - Acquisition Parameters
Date_ 20260524
Time 10.42 h
INSTRUM spect
PROBHD Z104450_0346 (
PULPROG hsqcetgp
TD 2048
SOLVENT CDCl3
NS 2
DS 0
SWH 8802.817 Hz
FIDRES 8.596501 Hz
AQ 0.1163264 sec
RG 2050
DW 56.800 usec
DE 6.50 usec
TE 158.4 K
CNST2 145.0000000
D0 0.00000300 sec
D1 1.00000000 sec
D4 0.00172414 sec
D11 0.03000000 sec
D16 0.00020000 sec
IN0 0.00001800 sec
TDav 1
ZGOPTNS
SFO1 400.1324708 MHz
NUC1 1H
P1 15.00 usec
P2 30.00 usec
PLW1 9.69999981 W
SFO2 100.6248425 MHz
NUC2 13C
CPDPRG[2 garp
P3 10.00 usec
P4 20.00 usec
PCPD2 80.00 usec
PLW2 47.00000000 W
PLW12 0.73438001 W
GPNAM[1] SINE.100
GPZ1 80.00 %
GPNAM[2] SINE.100
GPZ2 20.10 %
P16 1000.00 usec

F1 - Acquisition parameters
TD 217
SFO1 100.6248 MHz
FIDRES 256.016388 Hz
SW 276.053 ppm
FnMODE Echo-Antiecho

F2 - Processing parameters
SI 2048
SF 400.1300000 MHz
WDW QSINE
SSB 2
LB 0 Hz
GB 0
PC 1.40

F1 - Processing parameters
SI 1024
MC2 echo-antiecho
SF 100.6127690 MHz
WDW QSINE
SSB 2
LB 0 Hz
GB 0
